# The semantics of gaze in person perception: a novel qualitative-quantitative approach

**DOI:** 10.1038/s41598-024-51331-0

**Published:** 2024-01-09

**Authors:** Eva Landmann, Christina Breil, Lynn Huestegge, Anne Böckler

**Affiliations:** Department of Psychology, Julius-Maximilians-Universität Würzburg (JMU), 97070 Würzburg, Germany

**Keywords:** Human behaviour, Psychology

## Abstract

Interpreting gaze behavior is essential in evaluating interaction partners, yet the ‘semantics of gaze’ in dynamic interactions are still poorly understood. We aimed to comprehensively investigate effects of gaze behavior patterns in different conversation contexts, using a two-step, qualitative-quantitative procedure. Participants watched video clips of single persons listening to autobiographic narrations by another (invisible) person. The listener’s *gaze behavior* was manipulated in terms of gaze direction, frequency and direction of gaze shifts, and blink frequency; e*motional context* was manipulated through the valence of the narration (neutral/negative). In Experiment 1 (qualitative-exploratory), participants freely described which states and traits they attributed to the listener in each condition, allowing us to identify relevant aspects of person perception and to construct distinct rating scales that were implemented in Experiment 2 (quantitative-confirmatory). Results revealed systematic and differential meanings ascribed to the listener’s gaze behavior. For example, rapid blinking and fast gaze shifts were rated more negatively (e.g., restless and unnatural) than slower gaze behavior; downward gaze was evaluated more favorably (e.g., empathetic) than other gaze aversion types, especially in the emotionally negative context. Overall, our study contributes to a more systematic understanding of flexible gaze semantics in social interaction.

## Introduction

When it comes to forming impressions of our interaction partners, ‘reading’ their faces is a valuable nonverbal tool to gain insights into their attitudes, emotions, and intentions. For decades, research has uncarved the neural underpinnings^1–5^, moderators^6–9^, inter-individual differences^10,11^, and development^12–15^ of our astounding ability to recognize and classify faces. In studies on face perception, as well as person perception more generally, attributes like warmth and competence have been identified as key dimensions for judgments about others^16–19^. While the vast majority of these studies used *static* pictures, Pitcher and Ungerleider proposed the existence of a visual pathway specifically dedicated to the processing of *moving* faces and bodies^20^. Similarly, recent empirical evidence and theorizing suggest that a comprehensive understanding of how social signals are processed and interpreted requires experimental setups that capture the dynamic features of social interactions^21–23^. Eye gaze is one of the inherently dynamic biological stimuli presumably processed through the third pathway and is known to play a significant role in social interactions^24–26^. In the following, we provide a short overview of findings on core features of gaze behavior, namely *gaze direction* and the *frequency of changes* in gaze behavior*,* as well as on the moderating role of situational *context*.

*Gaze direction* is a particularly powerful parameter, with averted gaze inducing reflexive attention shifts^27^ and eye contact catching attention, supporting processing of social information, and inducing enhanced activation of several areas of the ‘social brain’^28–30^. An extensive body of research establishes direct gaze as an ultimate signal that affects how we interact with each other^24,25,30,31^. Humans are sensitive to being looked at from birth^32,33^ and quickly detect direct gaze in others^30^. Direct gaze is indicative of another’s interest in us and it shapes our evaluation of that person, leading us to perceive them as more attractive, likable, and trustworthy^34–36^. Given this generally positive evaluation of direct gaze, it could be assumed that any deviation of gaze results in less favorable evaluations of a person. Briefly averting one’s gaze can indicate that something or someone in the environment is capturing the person’s attention more than their interaction partner^24^ and may signal disinterest. Longer lasting avoidance of direct gaze has been reported to communicate rejection and social exclusion^37–39^ and can occur in the context of emotions such as shame and embarrassment^40^. Critically, initial evidence suggests that the categorization of gaze into ‘direct’ and ‘averted’ might not be sufficient, as the interpretation of averted gaze can vary depending on *where* the gaze is directed^41,42^. For instance, downward gaze (but not sideward gaze) can be associated with sadness^43,44^.

In addition to gaze direction, person perception is influenced by the *frequency of changes* in gaze behavior and the resulting interruptions in eye contact, such as through blinking. Individuals who blink at a rate that is consistent with the average human blink rate (approximately 15 to 20 blinks per minute^45,46^) appear to be perceived as particularly friendly^47,48^. Rapid blinking is often viewed unfavorably and may indicate nervousness or confusion, while slow blinks appear to signal intelligence and understanding^47–49^. Similar effects apply to the temporal dynamics of gaze direction changes as less frequent gaze shifts tend to be perceived more positively^50^. At the same time, however, eye contact becomes less pleasant when it exceeds 3–4 seconds^51^, suggesting that occasional gaze shifts are favorable in ongoing interactions. Importantly, recent studies show that situational factors also shape the appropriateness of sustained gaze^34^, highlighting that the interaction context needs to be considered when studying gaze behavior.

Specifically, research on *situational context* indicates that the processing of gaze behavior is influenced by cultural background^52–54^ and is dependent on the demands of the task at hand^55^. For instance, during conversations, eye contact varies depending on the roles of conversation partners^25,56^, with individuals typically making more eye contact with their interaction partner when they are listening (vs. speaking). Emotional context can also moderate social perception, for example, interpretations of facial expressions^57,58^ and attentional capture by direct gaze^59,60^. With regard to the evaluation of gaze in conversations, persons tend to look away in emotionally charged situations since this allows them to regulate their emotions or give the other person space to cope with theirs^25^. Accordingly, a recent study from our lab showed that in conversations on neutral topics, direct gaze was viewed as most trustworthy and empathetic. However, in emotionally negative contexts, occasional downward gaze shifts were more acceptable and, in some cases, even rated more favorably than uninterrupted eye contact^34^.

The present study was designed to systematically investigate how core features of gaze behavior (including, but not limited to gaze direction) and its emotional context influence person perception in dynamic conversation settings. To gain an extensive and *unbiased* understanding of gaze-related state and trait judgments, we implemented a two-step methodology. Experiment 1 identified relevant dimensions of person perception using an open-ended response format. While some previous theoretical frameworks a priori assume a fixed set of key dimensions in social evaluation^17,61^, we deliberately allowed participants to generate spontaneous gaze-associated semantics. In Experiment 2, rating scales were derived from the attributes mentioned in Experiment 1 to systematically quantify the evaluation of different core features of gaze behavior. In both experiments, we presented short video clips of a listener during a conversation and extensively manipulated the listener’s gaze behavior in terms of gaze direction, direction and frequency of gaze shifts, and blink frequency (see Table 1). By using videos of socially embedded conversations rather than static images, we captured the highly dynamic nature of actual social interactions. To account for the context-dependence of social signals, we manipulated the emotional valence of the conversation in the videos by having the short narrative (that the listener supposedly heard) deal with either neutral (e.g., work routines, daily events) or emotionally negative content (e.g., experiences of loss, illness, or failure)^34,62^. Examples for both neutral and negative narrations are provided in the Supplementary Materials (S1).

**Table 1:** Overview of factors and conditions of gaze behavior.

Block	Condition	Gaze behavior			Core aspects/Analyses
		Blink frequency	Gaze direction	Shift frequency	
Blink frequency	Blink 2 s	Fast	Direct	–	*Blink frequency* Exp1: Cochran’s Q tests Exp 2: 2 × 3 ANOVA
	Blink 4 s	Medium	Direct	–	
	Blink 6 s	Slow	Direct	–	
Constant gaze direction	Constant down	Medium	Down	–	*Gaze direction* Exp1: Cochran’s Q tests Exp2: 2 × 3 ANOVA
	Constant side	Medium	Side	–	
	Constant up	Medium	Up	–	
Shift frequency downwards	Shift down 2 s	Medium	Direct—down	Fast	*Shift frequency* */* *Shift direction* Exp1: Cochran’s Q tests Exp2: 2 × 3 × 3 ANOVA
	Shift down 4 s	Medium	Direct—down	Medium	
	Shift down 6 s	Medium	Direct—down	Slow	
Shift frequency sidewards	Shift side 2 s	Medium	Direct—side	Fast	
	Shift side 4 s	Medium	Direct—side	Medium	
	Shift side 6 s	Medium	Direct—side	Slow	
Shift frequency upwards	Shift up 2 s	Medium	Direct—up	Fast	
	Shift up 4 s	Medium	Direct—up	Medium	
	Shift up 6 s	Medium	Direct—up	Slow	

*ANOVA* analysis of variance.

All ANOVAs were conducted with the two-level factor emotional valence, while the respective gaze behavior was included as three-level factor. The factors shift frequency and shift direction were fully crossed and therefore included in a joint analysis.

We anticipated to replicate some previous findings, such as frequent (vs. infrequent) blinking and gaze shifting being rated less favorably^47–50^ and downward gaze being associated with sadness and empathy^43,44^, especially in emotionally negative contexts^34^. Critically, our study aimed to substantially expand previous findings by considering a more comprehensive (and unbiased) range of evaluative dimensions regarding numerous distinct features of gaze behavior. Given the multiplicity of our conditions and rating scales, we generated hypotheses based on the observations of Experiment 1 and focused our interpretation mainly on findings that were corroborated or specified in Experiment 2.

## Results

In two experiments, participants evaluated listeners who showed various forms of *gaze behavior* during conversations with different *emotional valence*. In Experiment 1, we examined the frequency with which attributes relating to (inductively formed) semantic categories were mentioned by participants in free-text responses (termed ‘frequency of mention’ hereafter). In Experiment 2, we analyzed participants’ ratings on scales reflecting the categories we identified in Experiment 1 (see Table 2).

**Table 2:** Inductively formed categories (Exp. 1) and corresponding rating scales (Exp. 2).

	Dimension	Exp. 1 categories	Exp. 2 rating scales
Bipolar categories	Attentiveness	Attentive	Inattentive—attentive
		Inattentive	
	Calmness	Calm	Restless—calm
		Restless	
	Empathy	Empathetic	Indifferent—empathetic
		Indifferent	
	Interest	Interested	Disinterested—interested
		Disinterested	
	Likability	Likable	Unlikable—likable
		Unlikable	
	Naturalness	Natural	Unnatural—natural
		Unnatural	
	Openness	Open	Avoidant—open
		Avoidant	
Unipolar categories	Annoyance	Annoyed	Not at all—extremely annoyed
	Bewilderment	Bewildered	Not at all—extremely bewildered
	Sadness	Sad	Not at all—extremely sad
Not included in Exp. 2	Amusement	Amused	–
	Anxiousness	Anxious	–
	Pensiveness	Pensive	–

In Experiment 2, 14 of the 20 categories from Experiment 1 were transformed into 7 bipolar scales, while 3 categories were included without semantic counterpart. All rating scales were presented as seven-point Likert scales. 3 categories were not included in Experiment 2 due to overall low numbers of attribute occurrence and a lack of differential effects in Experiment 1.

### Frequency of mentions of semantic categories

**Figure 1:**
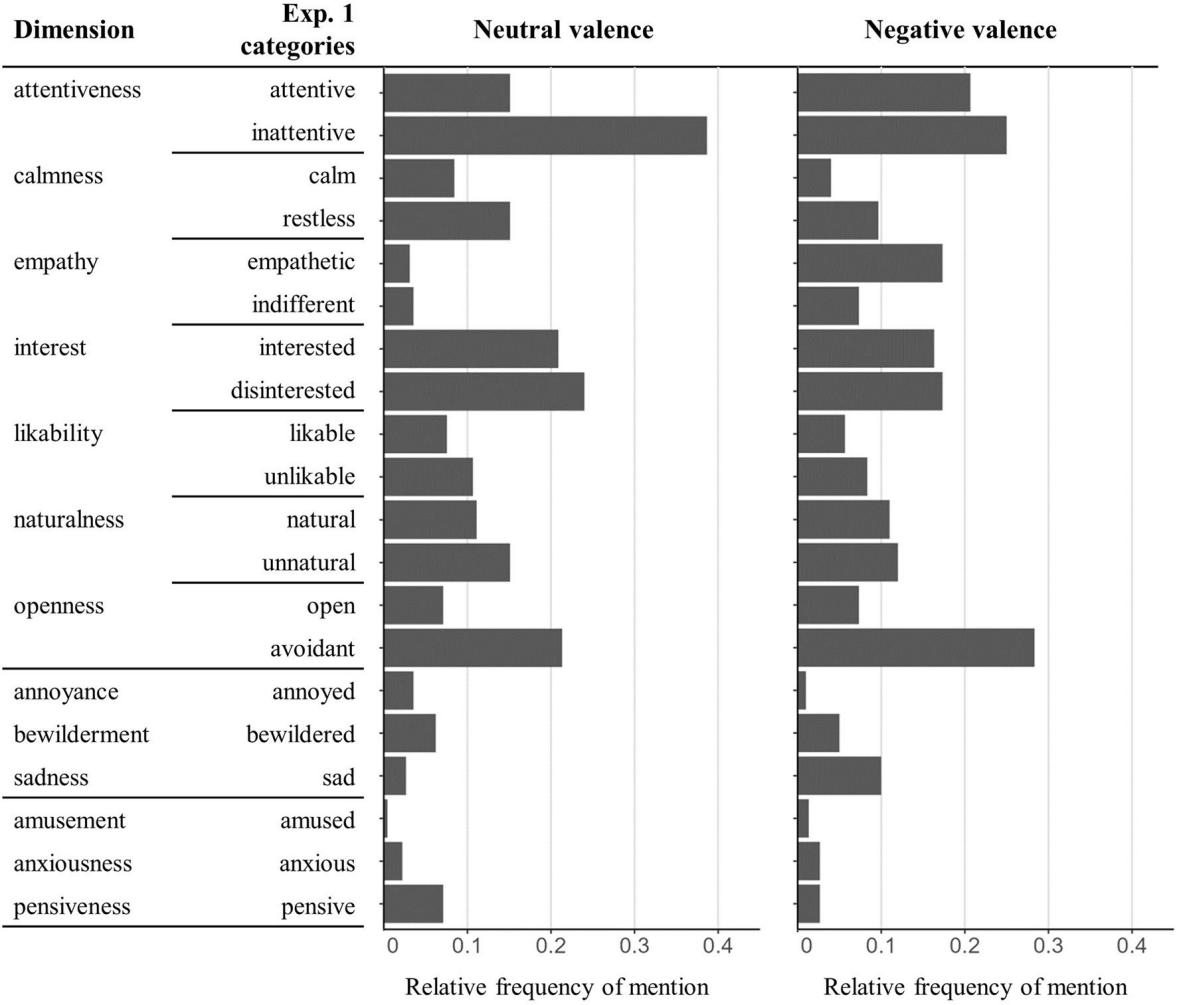
Relative frequencies of mention (Exp. 1): Number of references to the 20 semantic categories relative to the total number of free-text responses in the neutral (left side, n = 15, 225 responses) and the negative valence group (right side, n = 20, 300 responses).

A complete overview of the frequencies of mention in Experiment 1 is provided in Fig. 1. Participants in the neutral valence group most frequently mentioned attributes related to the semantic categories ‘inattentive’ (87 times), ‘disinterested’ (54 times), and ‘avoidant’ (48 times). In the negative valence group, attributes in the categories ‘avoidant’ (85 times), ‘inattentive’ (75 times) and ‘attentive’ (62 times) were mentioned most frequently. Notably, the categories ‘sad’ and ‘empathetic’ were rarely referred to in the neutral valence group (6 and 7 times, respectively), while they were frequently used to describe the listener in the negative valence condition (30 and 52 times, respectively).

### Differential effects of core features of gaze behavior

Results will be reported sequentially for the core manipulations of gaze behavior (blink frequency, gaze direction, frequency and direction of gaze shifts). In each section, we first describe results of Experiment 1, focusing on categories in which one condition significantly differed from the other conditions in terms of the number of participants mentioning at least one attribute related to the respective category (see Supplementary Tables S2–S5 for a complete overview of the results). Subsequently, results of Experiment 2 are reported focusing primarily on the main effects which pertained to observations of Experiment 1 (see Table 3 for a comprehensive overview of central results in Experiment 1 and 2). While the presented results of the analyses of variance (ANOVAs) are unadjusted for multiple comparisons, implementation of a conservative correction to account for the 10 analyses per core feature revealed that all but one reported main effect remained statistically significant. The supplementary materials provide a full overview of the corrected analyses along with further details, including all significant interactions and pairwise *t*-tests (S3.2, Supplementary Tables S6–S11). Finally, we report data from principal component analyses (PCA) which we conducted separately for each experimental condition in Experiment 2.

**Table 3:** Overview of categories with significant findings in Experiment 1 and 2.

Gaze behavior		Affected categories (Exp. 1)	Affected ratings (Exp. 2)
Blink frequency	Fast blinking (vs. medium/slow)	More inattentive and restless	Less attentive and calm
			*Add.*: More annoyed, bewildered, less interested^a^, natural, and open
	Medium blinking (vs. fast/slow)	More likable and natural	–
Constant gaze direction	Downward gaze (vs. side-/upward)	More empathetic^+^ and sad^+^	More empathetic^c^ and sad
			*Add.*: Less annoyed, more likable and natural
	Sideward gaze (vs. down-/upward)	More inattentive and less sad	Less attentive
			*Add.*: Less interested and open
	Upward gaze (vs. down-/sideward)	More annoyed	–
Shift frequency	Fast shifts (vs. medium/slow)	Less attentive, calm, interested, likable, natural, more avoidant and restless	Less attentive, calm, interested, natural^b^, open
			*Add.*: More annoyed, bewildered, less empathetic
	Slow shifts (vs. fast/medium)		*Add.*: Less annoyed and bewildered, more attentive, calm, empathetic, interested, likable, natural^b^, open
Shift direction	Downward shifts (vs. side-/upward)	More natural^+^	More natural
			*Add.*: Less annoyed, more attentive^c^, empathetic^c^, interested^c^, likable^c^, open, sad^c^
	Sideward shifts (vs. down-/upward)	More open	–
			*Add.*: Less calm
	Upward shifts (vs. down-/sideward)	More disinterested	–
			*Add.*: Less natural

*Add.:* Additional effects in Exp. 2 beyond observations from Exp. 1

^+^Descriptive effects that did not reach the criterion of significance.

^a^Not all pairwise comparisons significant.

^b^Significant interaction with valence (Exp. 2): Effect of gaze behavior more pronounced in neutral context.

^c^Significant interaction with valence (Exp. 2): Effect of gaze behavior more pronounced in negative context.

#### Blink frequency

**Figure 2:**
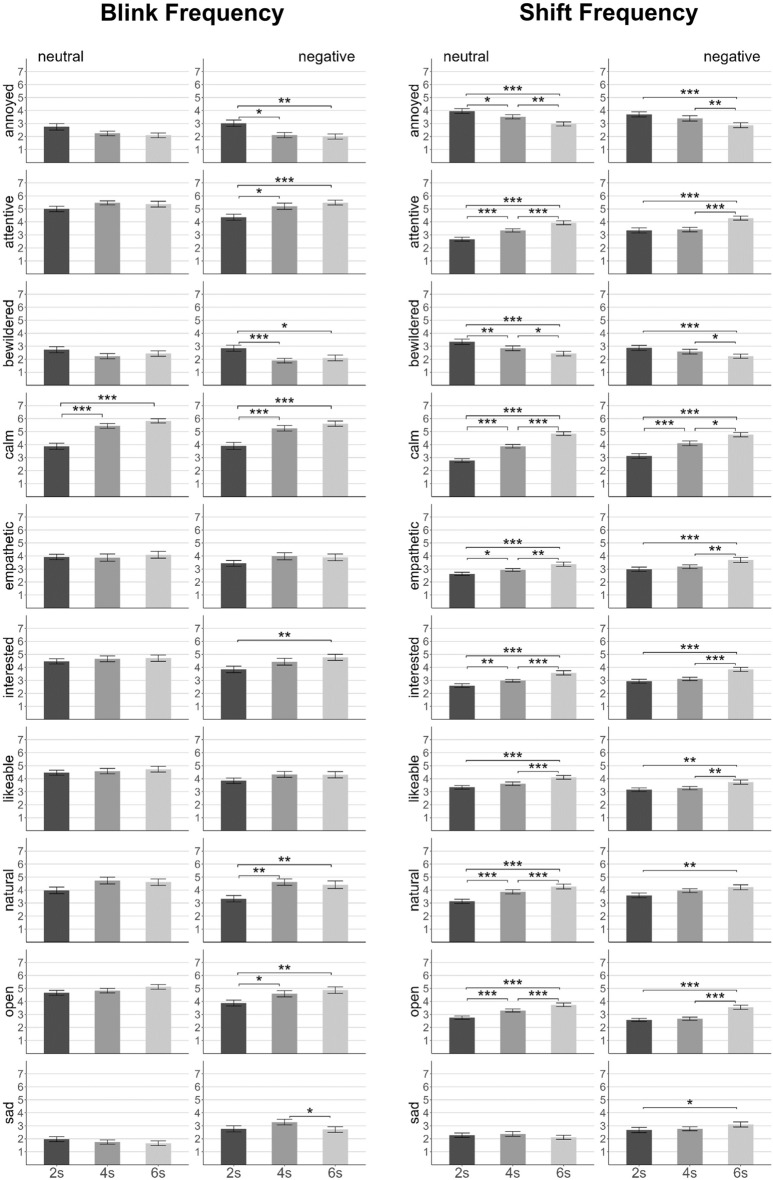
Mean ratings (Exp. 2) as a function of blink frequency (left side) and shift frequency (right side). Categories are presented in alphabetical order. Dark gray columns: fast frequency, medium gray columns: medium frequency, light gray columns: slow frequency. Error bars indicate standard errors. Horizontal bars indicate significant pairwise comparisons: *p < 0.05, **p < 0.01, ***p < 0.001.

The factor blink frequency entailed three conditions in which the listener displayed direct gaze while blinking fast (every 2 s), according to the average human blink rate (medium, every 4 s), or slowly (every 6 s). Blink frequency was analyzed using Cochran’s Q tests on all semantic categories with a liberal α of 0.10 to avoid missing potentially relevant effects. In Experiment 2, we performed 2 × 3 mixed ANOVAs (with α set to 0.05) on each rating scale with the between-subject factor emotional valence (neutral vs. negative) and the within-subjects factor blink frequency (2 s vs. 4 s vs. 6 s) (Fig. 2).

In *Experiment 1*, the fast blinking condition led to significantly more frequent mentions of attributes related to the categories ‘inattentive’ (only neutral group: Χ^2^(2) = 6.00, *p* = 0.050) and ‘restless’ (neutral: Χ^2^(2) = 12.67, *p* = 0.002; negative: Χ^2^(2) = 12.29, *p* = 0.002) compared to the other conditions. In the medium-fast blinking condition, more participants referred to attributes in the categories ‘likable’ (only neutral group: Χ2(2) = 8.00, *p* = 0.018) and ‘natural’ (only neutral group: Χ^2^(2) = 7.75, *p* = 0.021) than for the other blink frequencies.

In line with Experiment 1, results from *Experiment 2* suggested unfavorable evaluations of fast blinking. The main effect of blink frequency reached significance for the attributes ‘attentive’ (*F*(2,156) = 11.35, *p* < 0.001, ${\eta }_{p}^{2}$=0.13) and ‘calm’ (*F*(2,156) = 49.97, *p* < 0.001, ${\eta }_{p}^{2}$=0.39). Pairwise comparisons showed significantly lower ratings in the fast blinking compared to the other two conditions but no significant difference between medium-fast and slow blinking. Beyond replications of Experiment 1, we found a similar pattern of less favorable evaluations of fast blinking for ‘annoyed’ and ‘bewildered’ (i.e., significantly higher ratings in the fast blinking condition) as well as for ‘interested’, ‘natural’, and ‘open’ (i.e., significantly lower ratings in the fast blinking condition), all *F*s > 5.40, all *p*s < 0.008. In contrast to Experiment 1, the medium and slow frequency conditions did not significantly differ. Although ‘natural’ had the descriptively highest ratings in the medium-fast blinking condition, pairwise comparisons revealed no significant difference between medium and slow blinking. Similarly, there was no significant difference regarding the category ‘likable’ (*F*(2,156) = 2.75, *p* = 0.068, ${\eta }_{p}^{2}$=0.03).

#### Constant gaze direction

**Figure 3:**
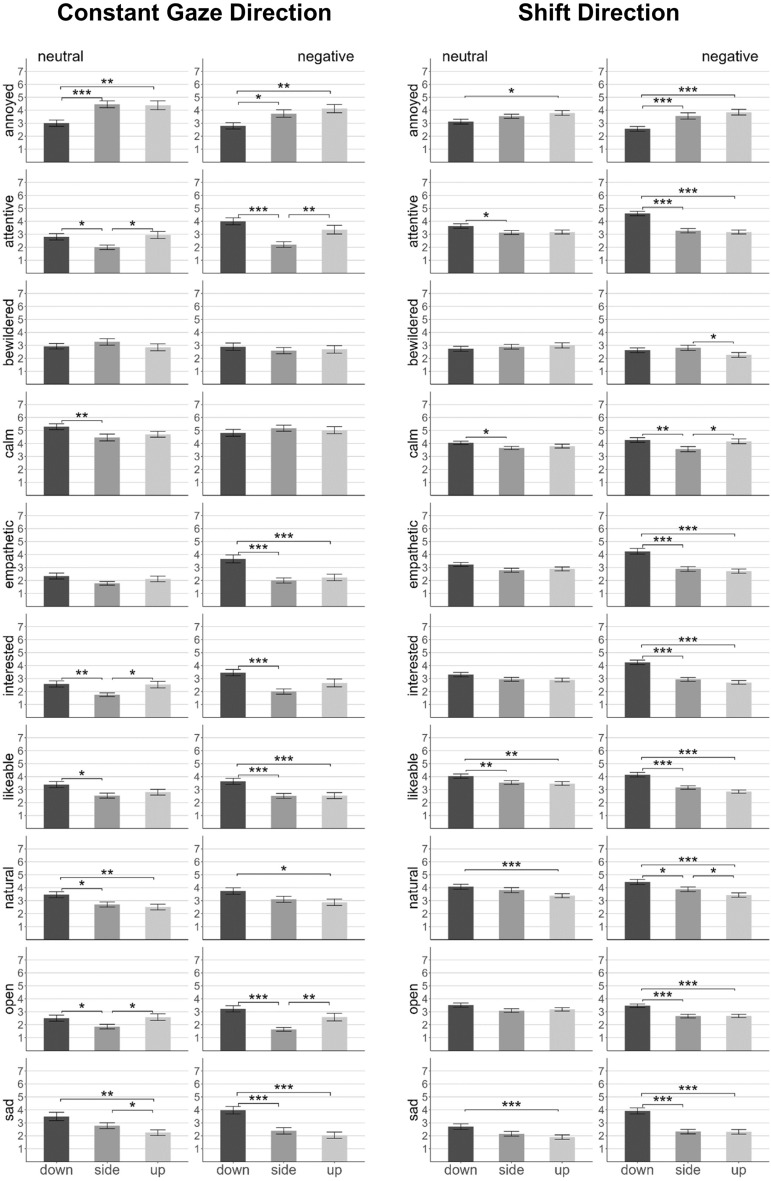
Mean ratings (Exp. 2) as a function of constant gaze direction (left side) and shift direction (right side). Categories are presented in alphabetical order. Dark gray columns: downward gaze, medium gray columns: sideward gaze, light gray columns: upward gaze. Error bars indicate standard errors. Horizontal bars indicate significant pairwise comparisons: *p < 0.05, **p < 0.01, ***p < 0.001.

Gaze direction entailed three conditions in which the listeners constantly averted their gaze either downwards, sidewards, or upwards. Similar to the analyses of blink frequency we conducted Cochran’s Q tests for Experiment 1 and 2 × 3 mixed ANOVAs for Experiment 2 (Fig. 3).

In *Experiment 1,* more participants mentioned attributes related to the category ‘inattentive’ (only negative group: Χ^2^(2) = 8.86, *p* = 0.012) and fewer participants mentioned attributes related to the category ‘sad’ (only negative group: Χ^2^(2) = 6.33, *p* = 0.042) for sidewards averted gaze compared to the other gaze directions. The upward gaze condition was associated with a higher frequency of mentions of attributes in the category ‘annoyed’ (only neutral group: Χ^2^(2) = 6.00, *p* = 0.050). In the downward gaze condition, the frequency of mention did not significantly differ from both other gaze directions for any of the semantic categories. However, when examined descriptively, mentions of attributes in the categories ‘empathetic’ and ‘sad’ were highest in the downwards gaze condition.

Matching and further clarifying the descriptive findings of Experiment 1, downward gaze was rated as more compassionate in *Experiment 2*: The main effects for ‘sad’ and ‘empathetic’ were significant (*F*(2,156) = 33.70, *p* < 0.001, ${\eta }_{p}^{2}$=0.30 and *F*(2,156) = 17.90, *p* < 0.001, ${\eta }_{p}^{2}$=0.19), with higher ratings in the downward gaze condition compared to the other gaze directions. A significant interaction with the between-factor emotional valence (*F*(2,156) = 6.05, *p* = 0.003, ${\eta }_{p}^{2}$=0.07) revealed that the significant effect of gaze direction for ‘empathetic’ was only present in the negative valence condition. Suggesting a generally more positive evaluation of downwards gaze, ‘likable’ and ‘natural’ (all *F*s > 10.37, all *p*s < 0.001) were rated significantly higher in the downward (vs. side-/upward) gaze condition and ‘annoyed’ (*F*(2,156) = 19.10, *p* < 0.001, ${\eta }_{p}^{2}$=0.20) was rated significantly lower in the downward (vs. side-/upward) gaze condition. In contrast to Experiment 1, upwards gaze did not elicit particularly high ratings for ‘annoyed’, as there was no significant difference between the side- and the upward gaze condition. Consistent with Experiment 1, sideward gaze led to significantly lower ratings for ‘attentive’ than the down- and upward gaze condition (*F*(2,156) = 17.18, *p* < 0.001, ${\eta }_{p}^{2}$=0.18). Similarly, and beyond the findings of Experiment 1, ratings were significantly lower for sidewards averted gaze compared to the other two conditions for ‘interested’ and ‘open’ (all *F*s > 14.88, all *p*s < 0.001).

#### Frequency of gaze shifts

Gaze shifts were manipulated by two factors, *frequency* of gaze shifts with fast (every 2 s), medium-fast (every 4 s) or slow (every 6 s) shifts and *direction* of gaze shifts with shifts from direct gaze to downward, sideward, or upward gaze. To analyze effects of gaze shift *frequency* in Experiment 1, we assessed whether a category was mentioned by participants in at least one of the respective conditions (across gaze shift directions) and conducted Cochran’s Q tests. In Experiment 2, we ran 2 × 2 × 3 mixed ANOVAs with the between-subject factor emotional valence (neutral vs. negative) and the within-subjects factors shift frequency (2 s vs. 4 s vs. 6 s) and shift direction (down vs. side vs. up) (Fig. 2).

In *Experiment 1,* attributes related to the following categories were mentioned less frequently in the fast (vs. the other two) shift condition: ‘attentive’ (neutral: Χ^2^(2) = 7.80, *p* = 0.020, negative: Χ^2^(2) = 6.00, *p* = 0.050), ‘calm’ (only neutral group: Χ^2^(2) = 5.43, *p* = 0.066), ‘interested’ (only neutral group: Χ^2^(2) = 4.67, *p* = 0.097), ‘likable’ (only negative group: Χ^2^(2) = 7.71, *p* = 0.021), and ‘natural’ (only negative group: Χ^2^(2) = 5.25, *p* = 0.072). Accordingly, attributes in the categories ‘avoidant’ (only neutral group: Χ^2^(2) = 9.75, *p* = 0.008) and ‘restless’ (only negative group: Χ^2^(2) = 8.86, *p* = 0.012) were mentioned more frequently for fast (vs. the other) shift frequencies.

In line with Experiment 1, the ratings in *Experiment 2* increased with decreasing shift frequency for ‘attentive’ (*F*(2,156) = 39.02, *p* < 0.001, ${\eta }_{p}^{2}$=0.33), ‘calm’ (*F*(2,156) = 104.83, *p* < 0.001, ${\eta }_{p}^{2}$=0.57), ‘interested’ (*F*(2,156) = 37.10, *p* < 0.001, ${\eta }_{p}^{2}$=0.32), ‘natural’ (*F*(2,156) = 37.64, *p* < 0.001, ${\eta }_{p}^{2}$=0.33) and ‘open’ (*F*(2,156) = 44.21, *p* < 0.001, ${\eta }_{p}^{2}$=0.36). There was also a significant main effect for ‘likable’ (*F*(2,156) = 24.81, *p* < 0.001, ${\eta }_{p}^{2}$=0.24) due to significantly higher ratings for slow gaze shifts compared to the other two. Three additional categories showed significant main effects as ratings decreased with increasing shift frequency for ‘empathetic’ and increased for ‘annoyed’ and ‘bewildered’ (all *F*s > 24.94, all *p*s < 0.001).

#### Direction of gaze shifts

Finally, we focused on the *direction* in which the listeners shifted their gaze with varying frequency from direct gaze to down-, side-, or upwards. We assessed whether a category was mentioned by participants in one or more of the respective conditions (across gaze shift frequencies) and conducted Cochran’s Q tests to compare shift directions in Experiment 1. In Experiment 2 we looked at effects involving the factor shift direction in the 2 × 2 × 3 mixed ANOVAs (Fig. 3).

In *Experiment* 1, attributes in the category ‘open’ were referred to by more participants (only neutral group: Χ^2^(2) = 7.60, *p* = 0.022) for gaze shifts to the side (vs. the other directions). The upward shift condition was associated with a higher frequency of mentions referring to the category ‘disinterested’ (only negative group: Χ^2^(2) = 8.91, *p* = 0.012). Downwards averted gaze did not significantly differ from both other shift directions for any of the semantic categories. Examined descriptively, mentions of attributes related to the category ‘natural’ were most frequent in the downward shift condition.

In accordance with descriptive findings in Experiment 1 and especially with analyses of constant gaze direction, downward gaze shifts were perceived more favorably in *Experiment 2*. The main effect of shift direction reached significance for ‘natural’ (*F*(2,156) = 25.47, *p* < 0.001, ${\eta }_{p}^{2}$=0.25), with pairwise comparisons corroborating the highest ratings for downward gaze and the lowest ratings for upward gaze. In addition, ratings for ‘annoyed’ were significantly lower in the downward shift compared to the other two conditions (*F*(2,156) = 22.25, *p* < 0.001, ${\eta }_{p}^{2}$=0.22). Accordingly, ratings for ‘attentive’, ‘empathetic’, ‘interested’, ‘likable’, and ‘sad’ were significantly higher in the downward shift condition, especially in the negative valence group. This was reflected in significant main effects (all *F*s > 30.13, all *p*s < 0.001) and significant interactions with emotional valence (all *F*s > 5.35, all *p*s < 0.006) for these categories. Contrary to findings of Experiment 1, we did not observe the highest ratings for ‘open’ in the sideward (but in the downward) shift condition (*F*(2,156) = 15.48, *p* < 0.001, ${\eta }_{p}^{2}$=0.17). However, the sideward shift condition differed from both other conditions regarding the category ‘calm’ in terms of significantly lower ratings (*F*(2,156) = 8.60 *p* < 0.001, ${\eta }_{p}^{2}$=0.10). Regarding the hypothesis of particularly low ratings for the category ‘interested’ in the upward shift condition, we found ratings to be descriptively but not statistically lower for upward (vs. down-/sideward) shifts.

### Principal component analysis

To examine whether the ratings scales in Experiment 2 could be structured according to overarching categories, we conducted PCAs for each gaze behavior and emotional valence condition, resulting in 30 analyses. The Kaiser–Meyer–Olkin measure verified the sampling adequacy with values between 0.72 and 0.89 (‘middling’ to ‘meritorious’^63^). Extracting components with eigenvalues > 1 produced two-component solutions in 17 of the 30 analyses (neutral: 10, negative: 7) and three-component solutions in the remaining analyses (neutral: 5, negative: 8).

In all solutions, a strong first component emerged (proportion of variance: 33.14–54.94%), with high loadings of the attributes ‘interested’, ‘likable’ and ‘empathetic’ as well as ‘attentive’, ‘open’ and ‘natural’ (for an example see Table 4, for an overview of all results see Supplementary Tables S14 and S15). We labeled this component ‘engaged listening’. The second component (12.85–26.97%) was reflected in high positive loadings of ‘bewildered’ and ‘annoyed’ and high negative loadings of ‘calm’ in most of the solutions. We named this component ‘agitation’. For the third component (11.50–15.78%), no clear pattern emerged.

**Table 4:** Exemplary factor loadings of the PCAs (fast downward shift condition).

Attribute	Neutral		Negative	
	Comp 1	Comp 2	Comp 1	Comp 2
Annoyed	** − 0.73**	0.38	− 0.11	** − 0.75**
Attentive	**0.79**	0.18	0.43	0.45
Bewildered	− 0.19	**0.73**	0.22	** − 0.60**
Calm	**0.57**	0.04	− 0.37	**0.66**
Empathetic	**0.84**	0.11	**0.91**	0.01
Interested	**0.83**	0.09	**0.90**	0.10
Likable	**0.86**	− 0.01	**0.91**	0.05
Natural	**0.79**	− 0.21	**0.84**	− 0.10
Open	**0.83**	0.03	0.29	**0.68**
Sad	0.11	**0.90**	**0.85**	− 0.07

*Comp* component.

Factor loadings >|0.50| are highlighted in bold.

The reported pattern of factor loadings did not differ markedly between the two emotional valence groups, except for the category ‘sad’. In the neutral valence group ‘sad’ had high loadings on the second component (‘agitation’) in almost all conditions, while in the negative valence group ’sad’ loaded particularly highly on the first component (‘engaged listening’).

## Discussion

In two experiments using dynamic videos of people displaying various gaze patterns while listening to another person during conversations, we explored the semantics of gaze in relation to person perception. Our approach was deliberately broad and encompassed multiple core features of gaze behavior and different emotional contexts. Together with a two-step qualitative-quantitative procedure allowing for an unbiased extraction of semantics, this resulted in an exceptionally rich, yet coherent pattern of empirical findings (see Table 3) that may help to guide research projects in the future. To ensure scientific rigor, the discussion will mainly focus on findings that were supported by both experiments.

As expected, when participants rated the listeners on ten state/trait attributes for each experimental condition (Experiment 2), more effects of gaze behavior were revealed than for the spontaneous, self-generated attributes (Experiment 1). This was especially true for effects concerning the *direction* of constant gaze and gaze shifts. However, the overall pattern of findings was largely consistent across experiments, corroborating and significantly extending prior research^34,43,44,47–50^.

First, we observed that the *frequency* of changes in gaze behavior (both in blinks and gaze direction) systematically shaped evaluations of calmness and authenticity, with faster changes being consistently associated with greater restlessness, bewilderment, avoidance, and unnaturalness. Emotional context did not further moderate this effect. Nevertheless, it of course appears conceivable that in other contexts, for instance in group settings or conversations about happy topics, more animated gaze behavior is preferred over slow changes.

Secondly, we found a clear pattern of effects for gaze *direction* both for constantly averted gaze and for gaze shifts. While previous research mainly distinguished between direct and averted eyes^26,29,64^, our findings suggest that it is crucial to consider the specific direction of averted gaze. We show that listeners looking downward were perceived systematically more attentive, empathetic, natural, and sad than when gaze was averted to the side or upwards. This effect was strongly moderated by emotional context, with downwards gaze appearing particularly favorable during negative conversations. In accordance with this pattern, emotional valence of the conversation impacted how the attribute ‘sad’ was grouped in the PCAs. Sadness in the listener was interpreted as a sign of engaged listening in a conversation dealing with *negative* topics, but as agitation and unrest in a *neutral* context.

In general, our findings emphasize the benefits of a less restricted, unbiased approach and the importance of considering context when selecting attributes for the investigation of person perception. If we had solely relied on dimensions identified in previous studies, we would have missed critical information relevant to the current dynamic setting. For example, we identified distinct states and traits that go beyond the traditional dimensions of person perception, competence and warmth^16,17^. While some of the attributes in our experiments might be considered facets of or in line with these constructs (e.g., likability as ‘warm’ trait), many of the remaining states and traits (e.g., attentiveness and interest, naturalness, calmness, sadness) are particularly crucial in coordination, cooperation, and conversation.

The context-dependent nature of the effects we observed emphasizes that even though eye gaze is a basic social signal that systematically attracts and influences attention across species, ages, and cultures^65^, there cannot be *one* universal semantics of gaze. What gaze behaviors convey, heavily depends on the situation, the relationship, and the people involved—which has been convincingly demonstrated for other facial signals like emotion expressions, too^58^. Of course, this apparent flexibility of gaze semantics also limits the generalizability of our own findings to our specific methodological choices. For example, as we did not employ a fully crossed design, the interplay of different features of gaze behavior remains unclear (e.g., does blink frequency shape person perception depending on gaze direction?). Interactions between gaze shift frequency and shift direction (exploratory analyses, see Supplemental Material S3.3, Supplementary Tables S12, S13) suggest that it could be a promising endeavor to investigate these relationships systematically. The inclusion of more iconic gaze behaviors (e.g., eye rolls) as well as more naturalistic and engaging conditions (e.g., participants being actively involved in conversations or action coordination) could be gradual steps forward towards a more comprehensive understanding of gaze semantics^22^. Particularly promising might be the investigation of more diverse emotional contexts, such as incorporating positively valenced narrations. Positive affective empathy not only stands as the most prevalent form of empathic responses in daily life^66^, but there are also indications of a close association between positive emotions and perceived gaze direction^67,68^. For instance, findings by McCrackin and Itier (2021)^67^ suggest that direct gaze may specifically facilitate empathy with positive emotions. In addition, future research should extend our findings to more diverse participants, listeners, and narrators. For example, research suggests that face processing strategies such as attention allocation to the eyes as well as performance in facial identity and emotion recognition vary depending on whether the observed face is part of the ingroup or outgroup^69–73^. These factors could systematically shape the influence of gaze behavior on social evaluations, reflected, for instance, in more pronounced and fine-grained effects for ingroup faces.

Taken together, we believe that the present two-step qualitative-quantitative approach, which harvests the benefits of a bottom-up perspective, offers a valuable means of overcoming limitations imposed by research traditions and prior results, not only in our own field but also in other areas of research. By manipulating a wide range of distinct gaze behaviors (beyond direct versus averted gaze) in different conversation contexts, our study reveals a complex, but well-structured pattern of gaze semantics in person perception. While some of the results may seem intuitive and commonsense (e.g., slow blinking being perceived as calm), our systematic and comprehensive examination across diverse attributes provides a nuanced and well-founded basis for further research in this area. Especially the context-dependent nature of gaze semantics will be an important aspect to consider in both research and practical applications (e.g., for phone applications that aim to ‘correct’ gaze direction during video chats). Defining inappropriate or divergent gaze behavior solely based on the behavior itself may be insufficient and prevent an understanding of the intricacy and the potential miscommunications in social interactions. For instance, a highly rejection-sensitive individual might disregard an emotionally charged context and, therefore, misinterpret the downcast eye of an interaction partner as disinterest or social exclusion instead of a coping mechanism.

In conclusion, our study provides valuable insights into the complexities of gaze behavior and its impact on social evaluations by revealing a consistent pattern as well as the context-dependent nature of gaze semantics using an ecologically valid approach.

## Methods

### Experiment 1

#### Participants

We recruited 36 participants via the online research platform Prolific (www.prolific.co). Data from one participant could not be included in the analyses because they did not provide valid responses in any of the text boxes in the study. The final sample therefore consisted of 35 participants (21 male, 13 female, 1 non-binary) with a mean age of 31.6 years (*SD* = 13.2). Since Experiment 1 was primarily exploratory and focused on qualitative data, we did not conduct power analyses to determine sample size.

All participants gave informed consent and were financially compensated after completing the experiment. The procedures complied with the ethical standards of the 1964 Declaration of Helsinki regarding the treatment of human participants in research. The use of negatively valenced narrations in our study was the only aspect that presented potential ethical concerns. Since we had received approval from the local ethics committee for a previous study^34^ employing similar stimuli (Ethikkommission des Institutes für Psychologie der Humanwissenschaftlichen Fakultät der Julius-Maximilians-Universität Würzburg, GZEK 2020-57), no renewed ethical approval was necessary according to regulations for psychological studies in Germany.

#### Stimuli

**Figure 4:**
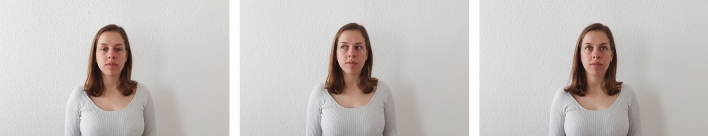
Recreated exemplary video stills of a listener in the constant gaze direction block. The listener’s gaze is averted downwards (left side), sidewards (middle) or upwards (right side).

We used four sets of 15 videos (resolution: 1920 × 1080 pixels), each set showing one of four persons introduced to the participant as ‘listener’. The featured listeners were young adults, two women and two men, and they were filmed in front of a neutral background, with their head and upper body visible. The listeners remained relatively still during the videos and maintained a neutral facial expression. Figure 4 displays recreated exemplary video stills featuring an individual who provided informed consent for publication of the images in an online open-access publication.

Within each listener’s set of videos, gaze behavior was systematically varied in terms of blink frequency, gaze direction, as well as frequency and direction of gaze shifts. These manipulations resulted in five experimental blocks, each consisting of three videos, in which exactly one aspect of gaze behavior was manipulated while the other aspects were kept constant (see Table 1). In the *blink frequency* block, the listener looked directly at the camera and blinked either fast (every 2 s), medium-fast (every 4 s), or slowly (every 6 s). In all other blocks, the listener blinked consistently every 4 s, in line with the average human blink rate. In the three videos of the *constant gaze direction* block, the listeners kept their gaze constantly averted either downward, to the side (i.e., from their perspective to the right), or upward.

In the remaining three blocks, frequency and direction of gaze shifts were systematically varied, and the listener switched gaze direction between looking directly at the camera and looking either downwards, sidewards, or upwards with varying frequency. In the *shift frequency down* block, the listener first looked directly at the camera for 2 (4, 6) seconds, then looked down for 2 (4, 6) seconds, then directly at the camera again, and so on. Similarly, in the *shift frequency side* block and the *shift frequency up* block, the listeners changed the direction of their gaze fast, medium-fast or slowly to the side or upwards, respectively.

Simultaneously with each video, an audio file was presented in which a narrator told a short autobiographical episode (in German) matched in length to the videos. The overall 30 audio narrations stemmed from 15 speakers (eight female and seven male) who each told two stories that differed in terms of their emotional valence. The neutral narrations entailed stories of work experience, hobbies, or social activities, whereas the stories of the second set dealt with negative topics such as illness, loss, and disappointment (see Supplement S1 for exemplary narratives). The narrations were drawn from the EmpaToM paradigm^62^, designed for the investigation of empathy and Theory of Mind. The manipulation of narrative valence as a key component of the EmpaToM procedure has been successfully implemented and validated in numerous studies^62,74–76^.

#### Design

Each participant saw only one listener throughout all 15 video conditions (split into five blocks à three videos). For half of the participants, the listener (and participant) only heard narrations with neutral content, for the other half, the listener heard only negatively valenced narrations. Hence, our study incorporated *gaze behavio*r of the listener as within-subject factor and *emotional valence* of the narration as between-subject factor. After data collection, group sizes were distributed somewhat unevenly, with 20 participants in the negative emotional valence group and 15 in the neutral valence group, which was taken into account in the analysis and interpretation of results.

#### Procedure

We used the Software Inquisit 6^77^ (version 6.5.1) for programming the study and running it online. Participants accessed the study link through their Prolific account, thereby preventing repeated participation. They had to install the Inquisit Player app on their device, which is supported for operating systems Windows 10 and Mac OS X 10.13.6 and higher on computers and laptops, as well as for Android 7.1 and iOS 11 and higher on mobile devices. Only two participants completed the experiment on a mobile device.

Participants first read the instructions, which transparently communicated the goal of the study, i.e., investigating the effect of different gaze behaviors on person perception. Participants were told that they would watch five blocks of three videos each, displaying a person listening to different autobiographic episodes told by different (invisible) narrators. They were asked to pay particular attention to the gaze behavior of the person listening and were encouraged to reflect and take notes after each video on the impressions they had formed.

After providing demographic data, the five blocks of the main task were presented in randomized order. At the beginning of each block, participants were transparently informed about the aspect in which the three videos differed (e.g., blink frequency) and were asked to note which gaze behavior was shown in which video. The order of the videos within the block was randomized. After the presentation of the three videos belonging to a block, participants were directed to a survey page with three open text boxes asking them to indicate which condition-specific characteristics, feelings, and intentions they would attribute to the listener in each of the corresponding videos. When they had filled in the text boxes, the next block began. After finishing all five blocks, participants were asked to indicate the strength of their motivation and diligence in completing the study (from ‘low’ to ‘very high’) before being redirected back to Prolific to receive their payment (results of these ratings are reported in the Supplementary Materials S2.1). The study took approximately 15 min to complete.

#### Analyses

To examine participants’ free-text responses, in a first step, two experts independently coded the attributes mentioned in each response and then clustered these attributes based on semantic similarity. The clusters were then compared and merged between experts, aiming for categories that were as clearly defined and internally consistent as possible (for an overview of coded attributes and semantic categories, see Supplementary Table S1). Of a total of 246 coded attributes, 15 were not categorized and analyzed because they were either mentioned extremely rarely (e.g., ‘overzealous’) or were not related to gaze behavior in any relevant way (for example the listener ‘having something in their eye’). The remaining attributes were inductively combined into 20 semantic categories.

In a second step, we coded for each semantic category whether it was mentioned (dichotomous: mentioned vs. not mentioned), separately for participants and experimental conditions. For each of the 20 semantic categories, we then examined effects of the four core manipulations of gaze behavior on the proportion of participants mentioning the respective category. First, we compared how often each of the categories was mentioned in the three videos which differed in terms of blink frequency (*blink frequency*: 2 s/4 s/6 s). Then, we compared the three videos differing with respect to the direction of constantly averted gaze (*constant gaze direction*: down/side/up) for all semantic categories.

The remaining comparisons involved the nine videos which manipulated frequency and direction of gaze shifts: First, these videos were compared regarding the *frequency of gaze shifts* (*shift frequency*: 2 s/4 s/6 s) across gaze shift directions by evaluating the proportion of participants mentioning the category in at least one of the respective conditions (e.g., for shift frequency 2 s: shift down 2 s, shift side 2 s, shift up 2 s). Finally, the same nine videos were compared regarding the *direction of gaze shifts* (*shift direction*: down/side/up) across gaze shift frequencies by assessing whether the category was mentioned by participants in at least one of the respective conditions (e.g., for shift direction down: shift down 2 s, shift down 4 s, shift down 6 s). Analyses were performed separately for responses from the neutral and negative emotional valence groups and were not directly compared between the groups due to unequal sample sizes.

We employed Cochran’s Q tests using R^78^ (version 4.2.1) with the package rstatix^79^ to determine whether the proportion of participants mentioning a certain category differed between the conditions of gaze behavior. As our primary objective at this stage was not to statistically quantify effects but to merely identify *potentially* relevant trends that could inform the subsequent quantitative experiment, we applied a liberal alpha level of 0.10. In cases in which we obtained significant results, we conducted pairwise McNemar tests between conditions. If one condition systematically differed from both other conditions (again α = 0.10), we converted the effect into an a priori hypothesis for Experiment 2. In the respective ‘results’ section, we only report analyses meeting this criterion.

## Experiment 2

### Participants

We recruited 80 new participants via Prolific (47 male, 33 female). The mean age in this sample was 30.3 years (*SD* = 9.0). To ensure that the study was presented correctly, only individuals using a PC or laptop could participate. All participants gave informed consent and were financially compensated after completion of the study. To assess the magnitude of effect size detectable with this sample size, we performed post-hoc sensitivity power analyses using G*Power^80^. Based on the mean correlation between repeated measures (*r* = 0.41) and mean Greenhouse–Geisser epsilon (ε = 0.92) observed in our data, we determined that with 80% power and an α of 0.05, the smallest effect size reliably discernible with our sample size was *f* = 0.16, indicating a small effect. This suggests that our statistical power was sufficient to identify relevant effects for our research interests.

#### Stimuli and rating scales

We utilized the same video and audio files as in Experiment 1, restricting the videos to a resolution of 854 × 480 pixels. Instead of open text boxes, we employed rating scales to measure participants’ evaluations of the listener. The scales were directly derived from the semantic categories formed in Experiment 1: Of the original 20 categories, 14 could be arranged into pairs of opposites (e.g., attentive/inattentive or likable/unlikable) and were combined into seven bipolar scales. The other six categories were considered unipolar (e.g., annoyed or bewildered). Three of these categories were not included in Experiment 2 as no trend (in terms of a difference between experimental conditions) was observed for them in Experiment 1, and they were also not frequently mentioned in any of the emotional valence groups (e.g., amused). Thus, we presented ten seven-point rating scales with verbally anchored ends, seven bipolar (e.g., disinterested—interested) and three unipolar (e.g., not at all sad—extremely sad).

#### Design

As in Experiment 1, gaze behavior was incorporated as within-subject factor and emotional valence as between-subject factor. The two valence conditions had slightly unequal group sizes, with 41 and 39 participants in the neutral and negative conditions, respectively. Participants were evenly distributed among the four ‘listeners’ in the videos. To control for order effects, the rating scales were counterbalanced, with each participant receiving one out of a total of ten possible orders, which were determined using a Latin square design.

#### Procedure

The procedure of Experiment 2 was largely similar to Experiment 1 and took about 25 min to complete. An explanation of the rating scales was added to the instructions, which also featured a sample scale for each category and the associated attributes that were most frequently mentioned for the respective category in Experiment 1 (serving as examples to understand the scale). To avoid a depletion of the participants’ attentional resources, the rating scales were divided into two blocks of five scales each. After watching the video once, participants completed the first five scales. They then watched the video again and completed the remaining five scales. At the end of each block, participants were presented with an overview of their ratings in this block and were asked to review their ratings. Here, they were given the opportunity to apply any corrections if they wished. After completing the task, participants indicated their level of motivation and diligence on a Likert scale (from ‘low’ to ‘very high’) (see Supplementary Material S3.1).

#### Analyses

As in Experiment 1, we evaluated the effect of four core manipulations of gaze behavior for each rated attribute using R^78^ (version 4.2.1), with the packages rstatix^79^ and afex^81^. To investigate the effect of blink frequency, we conducted 2 × 3 mixed-design ANOVAs comparing the three videos from the *blink frequency* block, with the between-subject factor emotional valence (neutral/negative) and the within-subject factor blink frequency (2 s/4 s/6 s). Similarly, we ran 2 × 3 mixed-design ANOVAs on the three videos from the *constant gaze direction* block, with emotional valence as the between-subject factor and gaze direction as the within-subject factor (down/side/up). For the nine videos from the *shift frequency down*, *shift frequency side* and *shift frequency up* blocks, we conducted 2 × 3 × 3 mixed-design ANOVAs with the between-subject factor emotional valence and the within-subject factors direction of gaze shifts (down/side/up) and frequency of gaze shifts (2 s/4 s/6 s). To account for sphericity violations, we applied Greenhouse–Geisser corrections to all ANOVAs. In the ‘Results’ section, we report corrected *p*s but uncorrected degrees of freedom for improved readability, while the Supplemental Tables S6 and S9–S12 provide an overview over the epsilons used for each correction.

In order to explore differences between gaze behavior conditions beyond main effects, we conducted pairwise *t*-tests between relevant conditions. To account for multiple comparisons, we applied Bonferroni correction to *t*-tests exploring the same main effect. In instances of significant interactions, we separately conducted ANOVAs for individual factor levels, such as for the neutral and negative groups. We report partial η^2^ and Cohen’s *d* as effect sizes.

Additionally, we aimed to explore whether the different semantic categories that participants had used to describe the listeners could be further organized into meaningful groups. Therefore, we performed Principal Component Analyses (using the R package ‘jmv’^82^) on the ten attributes reflected in the rating scales separately for each gaze behavior and emotional valence condition (30 PCAs overall). The analyses were conducted with oblimin rotation and we identified factors that had eigenvalues greater than 1.0.

### Supplementary Information

Supplementary Information.

## Data Availability

Raw data and analysis scripts for both experiments are available on the Open Science Framework repository (https://osf.io/38ke5).
