# Supplementary material for: The semantics of gaze in person perception: a novel qualitative-quantitative approach
Source: Sci Rep. 2024 Jan 9;14:893. doi: 10.1038/s41598-024-51331-0 (PMC10776783; doi:10.1038/s41598-024-51331-0)
Supplement: Supplementary file 1 — Supplementary Information. [file 41598_2024_51331_MOESM1_ESM.pdf]

Supplementary Materials for

The Semantics of Gaze in Person Perception: A Novel Qualitative-Quantitative Approach

Eva Landmann[1,*], Christina Breil[1], Lynn Huestegge[1,+], and Anne Böckler[1,+]

[1]Julius-Maximilians-Universität Würzburg (JMU), Department of Psychology, Würzburg, 97070, Germany
*eva.landmann@uni-wuerzburg.de
[+]these authors contributed equally to this work

This PDF file includes:

S1 Exemplary transcripts of narrations used in the neutral and negative emotional context condition

S2 Supplementary results for Experiment 1
- S2.1 Self-reported motivation and diligence of participants
- Table S1 Semantic categories derived from the freely associated attributes mentioned by the participants in Experiment 1
- Table S2 Analyses of blink frequency in Experiment 1
- Table S3 Analyses of constant gaze direction in Experiment 1
- Table S4 Analyses of shift frequency in Experiment 1
- Table S5 Analyses of shift direction in Experiment 1
- Figure S1 Relative frequency of mention for blink frequency and shift frequency
- Figure S2 Relative frequency of mention for constant gaze direction and shift direction

S3 Supplementary results for Experiment 2
- S3.1 Self-reported motivation and diligence of participants
- S3.2 Main effects of emotional valence and interactions between emotional valence and gaze behavior
- S3.3 Interactions between shift direction and shift frequency (exploratory analyses)
- Table S6 Main effects of gaze behavior in Experiment 2
- Table S7 Pairwise *t*-tests between conditions of gaze behavior in Experiment 2
- Table S8 Main effects of emotional valence in Experiment 2
- Table S9 Interactions between emotional valence and gaze behavior in Experiment 2
- Table S10 Main effects of gaze behavior in the neutral context group in Experiment 2
- Table S11 Main effects of gaze behavior in the negative context group in Experiment 2
- Table S12 Interactions between shift direction and shift frequency in Experiment 2
- Table S13 Main effects of shift frequency separated by shift direction
- Table S14 Overview of Principal Component Analyses
- Table S15 Factor loadings of all 30 Principal Component Analyses

S1 Exemplary transcripts of narrations used in the neutral and negative emotional context condition

All narrations used in the study were drawn from the EmpaToM paradigm (Kanske et al. 2015) and presented in German. The following examples have been translated to English by the authors.

**Neutral context**	**Negative context**
Yeah, so right now, I'm doing an apprenticeship as a photographer... just started recently. Everything's going great so far... also get along really well with my boss and all. Yeah, occasionally, I also work at weddings...	Somehow, for us, it was quite normal to take drugs. I had no idea that... I couldn't breathe anymore. In the hospital, they told me it was a heart attack!
I'm reading quite a lot at the moment. I'm looking at German classics... Thomas Mann and stuff like that... I enjoy it, um... I also need it for my studies. And um... yes, when I'm finished, I wanted to look at Russian literature in particular.	My father is an alcoholic. He often doesn't come home for days on end... and then the police bring him in. And my mother worries like crazy. But... I'm worried too... But I can't go home every time...
Yes, so I started playing chess when I was ten. I played my first tournament for school when I was 15. It all happened because of my sports teacher, who was a big chess fan, and he used to practice with me in the afternoons.	My daughter Sarah has epilepsy. She had her first seizure shortly after she was born, and I thought she was going to die. She's on medication, but I can't take my eyes off her.
I actually always sang in the choir at school. And now, for my studies, I had to move, so I looked for something here again. We rehearse a bit more frequently now, at least twice a week, and we also perform in a few concerts at the church.	I came out of the library pretty late, and he was already standing outside, not letting me pass... and he cornered me... I was paralyzed; if only I had done anything.
So, my little one, Tobias, is really good at handling snakes already. Of course, he brags about it in class. And I've already received concerned calls from teachers (smiles)... yeah, they think I'm letting him handle the snakes all by himself just because I'm not afraid of the animals!	I've had to draw my weapon a few times; I've been on patrol for several years. And last year, for the first time, I had to shoot for real... he bled out before the ambulance arrived.

S2 Supplementary results for Experiment 1

S2.1 Self-reported motivation and diligence of participants

After the completion of Experiment 1, participants were asked to rate their level of motivation and diligence in completing the study on a slider scale (range 1-9). In the sample, the average score was 7.1 (*SD*=1.4), and the ratings varied between 4 and 9.

Table S1: Semantic categories (left) derived from the freely associated attributes mentioned by the participants (right) in Experiment 1

Semantic category	Associated attributes (German)	Associated attributes (English translation)
attentive	aktiv zuhörend, andächtig, aufmerksam, eingenommen, fokussiert, gebannt, konzentriert, versuchtes zuhören, vertieft, zuhören, zuhören wollen	actively listening, devout, attentive, absorbed, focused, captivated, concentrated, attempting to listen, engrossed, listening, wanting to listen
inattentive	abgelenkt, ablenkbar, abschweifend, an anderes denken, auf anderes achten, kein zuhören, mental abwesend, mit sich beschäftigt, unaufmerksam, unfokussiert, unkonzentriert, zerstreut	distracted, distractible, digressing, thinking of other things, paying attention to other things, not listening, mentally absent, preoccupied, inattentive, unfocused, unconcentrated, scattered
calm	ausgeglichen, ausgeruht, entspannt, geduldig, gelassen, ruhig, zufrieden	balanced, rested, relaxed, patient, composed, calm, satisfied
restless	abwartend, aufgeregt, hektisch, nervös, unausgeglichen, ungeduldig, unruhig, zappelig, Zeit herumbringen, Zittern	waiting, agitated, hectic, nervous, unbalanced, impatient, restless, fidgety, wanting to pass the time, shaky
empathetic	anteilnehmend, berührt, betreten, betroffen, einfühlend, emotional, emotionales Coping, empathisch, mitfühlend, mitgenommen, taktvolles wegsehen, verständnisvoll, verstehen, zugeneigt	sympathetic, touched, concerned, empathizing, emotional, emotional coping, empathetic, compassionate, affected, tactfully looking away, understanding, understands, inclined
indifferent	apathisch, empathielos, gleichgültig, teilnahmslos, unbeeindruckt, unbeteiligt, wenig Anteilnahme, wenig Einfühlen, wenig Emotion, wenig Reaktion	apathetic, without empathy, indifferent, impassive, unimpressed, uninvolved, little sympathy, little empathy, little emotion, little reaction
interested	fasziniert, interessiert, mitdenken, neugierig, versuchtes Interesse	fascinated, interested, thinking along, curious, attempting interest
disinterested	desinteressiert, gelangweilt	disinterested, bored
likable	bequem, bestätigend, freundlich, höfliches zuhören, nett, nimmt ernst, respektvoll, sympathisch, unterstützend, vertrauenswürdig, vertraulich, wohlgesonnen	comfortable, affirming, friendly, polite listening, nice, takes seriously, respectful, sympathetic, supportive, trustworthy, confidential, well-meaning
unlikable	ablehnend, abschätzig, aggressiv, beleidigend, böse, dominant, einschüchternd, gereizt, gespieltes zuhören, ignorierend, nicht vertrauenswürdig, respektlos, störend, trotzig, unaufrichtig, unbequem, unecht, unehrlich, unhöflich, unsympathisch, verachtend, verärgert, verdächtig, wütend	dismissive, disparaging, aggressive, insulting, nasty, domineering, intimidating, irritable, fake listening, ignoring, untrustworthy, disrespectful, disruptive, defiant, insincere, uncomfortable, dishonest, rude, unlikable, contemptuous, angered, suspicious, angry

natural	angemessen, angenehm, authentisch, durchschnittlich, erfrischend, gut, menschlich, natürlich, neutral, normal, optimal, positiv, wenig Störung	appropriate, pleasant, authentic, average, refreshing, good, human, natural, neutral, normal, optimal, positive, little disturbance
unnatural	ablenkend, auffällig, beängstigend, befremdlich, besessen, beunruhigend, dümmlich, frustrierend, intensiv, komisch, krankhaft, schlecht, schrullig, seltsam, starr, starrend, steif, unangemessen, unangenehm, ungewöhnlich, unheimlich, unmenschlich, unnatürlich, verstörend, verunsichernd	distracting, conspicuous, alarming, alienating, obsessive, disturbing, inane, frustrating, intense, weird, pathological, bad, quirky, strange, rigid, staring, stiff, inappropriate, unpleasant, unusual, uncanny, inhuman, unnatural, disturbing, unsettling
open	aktiv, angeregt, direkt, dynamisch, entschlossen, fröhlich, froh, furchtlos, gesprächsbereit, hoffnungsvoll, Kontakt aufnehmen, offen, sachlich, selbstbewusst, sorgenfrei, überzeugt, wachsam, zuversichtlich	active, animated, direct, dynamic, purposeful, cheerful, joyful, fearless, ready to talk, hopeful, making contact, open, to the point, self-confident, carefree, convinced, alert, confident
avoidant	beschämt, bescheiden, Besseres zu tun, defensiv, dissoziiert, distanziert, flüchten wollen, Gefühle verbergen, Gespräch beenden wollen, Gespräch vermeiden wollen, in sich gekehrt, introvertiert, keine Interaktion, Kontakt vermeiden, müde, nicht beteiligen wollen, peinlich berührt, reuevoll, schüchtern, schuldig, sozial phobisch, submissiv, unangenehm empfinden, unsicher, unvertraulich, unwillig, unwohl, verlegen, verschlossen, verstört, wenig Emotionen zeigen, wenig Interesse zeigen, wenig Selbstbewusstsein, woanders sein wollen, zurückhaltend	ashamed, humbled, better things to do, defensive, dissociated, distant, wanting to escape, hiding feelings, wanting to end conversation, wanting to avoid conversation, introverted, no interaction, avoiding contact, tired, not wanting to participate, embarrassed, remorseful, shy, guilty, socially phobic, submissive, feeling uncomfortable, insecure, untrusting, unwilling, uneasy, abashed, reserved, distraught, showing little emotion, showing little interest, little self-confidence, wanting to be somewhere else, reserved
annoyed	Augenrollen, frustriert, genervt	Eye rolling, frustrated, annoyed
bewildered	fragend, geschockt, irritiert, kein verstehen, komisch finden, ratlos, überfordert, überrascht, ungläubig, verwirrt, verwundert	questioning, shocked, irritated, no comprehension, finding it strange, at a loss, overwhelmed, surprised, disbelieving, confused, astonished
sad	deprimiert, etwas auf dem Herzen, gramvoll, hoffnungslos, niedergeschlagen, resigniert, traurig, verletzt, verzweifelt	depressed, having something on the mind, sorrowful, hopeless, depressed, resigned, sad, hurt, desperate
amused	amüsiert, schelmisch, spielerisch	amused, mischievous, playful
anxious	ängstlich, eingeschüchtert, panisch, verängstigt	anxious, intimidated, panicked, scared
pensive	nachdenken, nachdenklich	contemplating, pensive

not included due to extremely rare mentions or irrelevance of content	angeekelt, angestrengt, auf Brust schauen, auf Körper schauen, enttäuscht, etwas im Auge, etwas sagen wollen, langsam, mit Augen verfolgen, mysteriös, präsent, übereifrig, uneindeutig, verwirrend, zu Sprecher hochsehen	disgusted, strained, looking at chest, looking at body, disappointed, something in eye, wanting to say something, slow, tracking with eyes, mysterious, present, overzealous, ambiguous, confusing, looking up at speaker

Table S2: Analyses of blink frequency in Experiment 1

**Blink frequency**	Frequency of mention			Cochran's Q test			*p*-values of pairwise McNemar tests		
**Neutral context**	2s	4s	6s	X²	df	*p*	2s vs. 4s	2s vs. 6s	4s vs. 6s
amused	1	0	0	2.00	2	0.368	0.317	0.317	-
annoyed	0	0	0	-	2	-	-	-	-
anxious	1	0	0	2.00	2	0.368	0.317	0.317	-
attentive	1	3	5	3.00	2	0.223	0.317	0.102	0.414
avoidant	4	0	1	5.20	2	0.074	0.046	0.180	0.317
bewildered	1	1	1	0.00	2	1.000	1.000	1.000	-
calm	0	3	1	4.67	2	0.097	0.083	0.317	0.157
disinterested	2	1	1	0.67	2	0.717	0.317	0.564	1.000
empathetic	0	2	1	2.00	2	0.368	0.157	0.317	0.564
inattentive	3	0	0	6.00	2	0.050	0.083	0.083	-
indifferent	0	1	1	1.00	2	0.607	0.317	0.317	1.000
interested	2	5	4	2.00	2	0.368	0.083	0.414	0.655
likable	0	4	0	8.00	2	0.018	0.046	-	0.046
natural	2	7	1	7.75	2	0.021	0.025	0.564	0.034
open	0	2	2	2.67	2	0.264	0.157	0.157	1.000
pensive	0	0	0	-	2	-	-	-	-
restless	8	1	0	12.67	2	0.002	0.020	0.005	0.317
sad	1	0	0	2.00	2	0.368	0.317	0.317	-
unlikable	0	1	3	3.50	2	0.174	0.317	0.083	0.317
unnatural	2	0	6	8.00	2	0.018	0.157	0.102	0.014
**Negative context**	2s	4s	6s	X²	df	*p*	2s vs. 4s	2s vs. 6s	4s vs. 6s
amused	1	0	1	2.00	2	0.368	0.317	-	0.317
annoyed	1	0	0	2.00	2	0.368	0.317	0.317	-
anxious	0	0	2	4.00	2	0.135	-	0.157	0.157
attentive	1	4	7	5.40	2	0.067	0.180	0.034	0.257
avoidant	5	1	5	4.57	2	0.102	0.046	1.000	0.046
bewildered	2	1	0	3.00	2	0.223	0.317	0.157	0.317
calm	0	2	1	2.00	2	0.368	0.157	0.317	0.564
disinterested	0	0	1	2.00	2	0.368	-	0.317	0.317
empathetic	1	5	3	3.43	2	0.180	0.046	0.317	0.414
inattentive	3	1	3	1.33	2	0.513	0.317	1.000	0.317
indifferent	2	5	1	4.33	2	0.115	0.083	0.564	0.102
interested	2	4	4	2.67	2	0.264	0.157	0.157	1.000
likable	0	1	0	2.00	2	0.368	0.317	-	0.317
natural	3	6	4	2.00	2	0.368	0.180	0.564	0.414
open	3	1	2	2.00	2	0.368	0.157	0.317	0.564
pensive	0	0	1	2.00	2	0.368	-	0.317	0.317
restless	7	0	1	12.29	2	0.002	0.008	0.014	0.317
sad	2	2	2	0.00	2	1.000	1.000	1.000	1.000
unlikable	3	1	1	2.00	2	0.368	0.317	0.157	1.000
unnatural	5	1	5	3.56	2	0.169	0.046	1.000	0.102

Frequency of mention: Number of participants mentioning at least one attribute referring to the respective semantic category.

Table S3: Analyses of constant gaze direction in Experiment 1

Constant gaze direction	Frequency of mention			Cochran's Q test			*p*-values of pairwise McNemar tests		
**Neutral context**	d	s	u	X^2	df	*p*	d vs. s	d vs. u	s vs. u
amused	0	0	0	-	2	-	-	-	-
annoyed	0	0	3	6.00	2	0.050	-	0.083	0.083
anxious	1	0	1	1.00	2	0.607	0.317	1.000	0.317
attentive	1	0	4	5.20	2	0.074	0.317	0.180	0.046
avoidant	6	5	3	1.40	2	0.497	0.705	0.257	0.414
bewildered	0	1	1	1.00	2	0.607	0.317	0.317	1.000
calm	1	0	1	2.00	2	0.368	0.317	-	0.317
disinterested	4	5	3	0.67	2	0.717	0.705	0.655	0.414
empathetic	1	0	0	2.00	2	0.368	0.317	0.317	-
inattentive	5	7	2	5.43	2	0.066	0.317	0.180	0.025
indifferent	1	0	1	1.00	2	0.607	0.317	1.000	0.317
interested	3	0	3	3.60	2	0.165	0.083	1.000	0.083
likable	2	0	0	4.00	2	0.135	0.157	0.157	-
natural	0	3	1	3.50	2	0.174	0.083	0.317	0.317
open	0	0	2	4.00	2	0.135	-	0.157	0.157
pensive	3	0	1	4.67	2	0.097	0.083	0.157	0.317
restless	1	2	2	2.00	2	0.368	0.317	0.317	-
sad	2	0	0	4.00	2	0.135	0.157	0.157	-
unlikable	1	4	1	3.60	2	0.165	0.083	1.000	0.180
unnatural	1	1	1	0.00	2	1.000	1.000	-	1.000
**Negative context**	d	s	u	X^2	df	*p*	d vs. s	d vs. u	s vs. u
amused	0	0	0	-	2	-	-	-	-
annoyed	1	0	1	1.00	2	0.607	0.317	1.000	0.317
anxious	0	0	0	-	2	-	0.317	1.000	0.317
attentive	4	2	8	7.00	2	0.030	0.317	0.102	0.014
avoidant	5	5	5	0.00	2	1.000	1.000	1.000	1.000
bewildered	1	1	1	0.00	2	1.000	1.000	1.000	1.000
calm	0	0	1	2.00	2	0.368	-	0.317	0.317
disinterested	2	8	6	4.67	2	0.097	0.058	0.102	0.480
empathetic	6	2	0	8.00	2	0.018	0.102	0.014	0.157
inattentive	2	7	1	8.86	2	0.012	0.059	0.317	0.014
indifferent	1	1	1	0.00	2	1.000	1.000	1.000	-
interested	2	0	4	4.80	2	0.091	0.157	0.317	0.046
likable	0	2	0	4.00	2	0.135	0.157	-	0.157
natural	1	0	2	3.00	2	0.223	0.317	0.317	0.157
open	1	1	1	0.00	2	1.000	1.000	1.000	1.000
pensive	1	1	0	1.00	2	0.607	1.000	0.317	0.317
restless	1	1	1	0.00	2	1.000	1.000	1.000	1.000
sad	5	0	3	6.33	2	0.042	0.025	0.317	0.083
unlikable	3	3	1	1.60	2	0.449	1.000	0.317	0.157
unnatural	2	2	1	0.67	2	0.717	1.000	0.317	0.564

Frequency of mention: Number of participants mentioning at least one attribute referring to the respective semantic category. d: downward, s: sideward, u: upward.

Table S4: Analyses of shift frequency in Experiment 1

Shift frequency	Frequency of mention			Cochran's Q test			*p*-values of pairwise McNemar tests		
**Neutral context**	2s	4s	6s	X^2	df	*p*	2s vs. 4s	2s vs. 6s	4s vs. 6s
amused	0	0	0	-	2	-	-	-	-
annoyed	1	1	1	-	2	-	-	-	-
anxious	2	0	0	4.00	2	0.135	0.157	0.157	-
attentive	0	5	7	7.80	2	0.020	0.025	0.008	0.480
avoidant	9	4	2	9.75	2	0.008	0.025	0.008	0.317
bewildered	2	2	2	0.00	2	1.000	1.000	1.000	1.000
calm	0	3	5	5.43	2	0.066	0.083	0.025	0.414
disinterested	8	8	5	1.80	2	0.407	1.000	0.257	0.257
empathetic	0	1	2	2.00	2	0.368	0.317	0.157	0.564
inattentive	10	10	8	2.67	2	0.264	1.000	0.157	0.157
indifferent	1	1	1	0.00	2	1.000	1.000	-	1.000
interested	3	7	8	4.67	2	0.097	0.046	0.059	0.705
likable	3	3	2	0.33	2	0.846	1.000	0.564	0.655
natural	2	4	3	1.00	2	0.607	0.157	0.655	0.655
open	0	1	4	5.20	2	0.074	0.317	0.046	0.180
pensive	3	2	3	0.50	2	0.779	0.564	1.000	0.317
restless	6	4	3	2.33	2	0.311	0.317	0.180	0.564
sad	2	1	0	3.00	2	0.223	0.317	0.157	0.317
unlikable	3	2	2	0.40	2	0.819	0.564	0.564	1.000
unnatural	2	3	5	2.80	2	0.247	0.564	0.083	0.317
**Negative context**	2s	4s	6s	X^2	df	*p*	2s vs. 4s	2s vs. 6s	4s vs. 6s
amused	0	1	0	2.00	2	0.368	0.317	-	0.317
annoyed	0	0	0	-	2	-	-	-	-
anxious	2	2	1	0.50	2	0.779	1.000	0.564	0.564
attentive	3	6	9	6.00	2	0.050	0.083	0.034	0.257
avoidant	10	10	8	0.73	2	0.695	1.000	0.527	0.527
bewildered	4	1	1	3.60	2	0.165	0.083	0.180	1.000
calm	0	4	2	6.00	2	0.050	0.046	0.157	0.157
disinterested	7	7	8	0.15	2	0.926	1.000	0.763	0.739
empathetic	4	7	7	2.25	2	0.325	0.257	0.180	1.000
inattentive	12	10	8	4.00	2	0.135	0.157	0.102	0.317
indifferent	3	2	1	1.50	2	0.472	0.317	0.317	0.564
interested	6	5	9	2.17	2	0.338	0.705	0.317	0.157
likable	0	3	6	7.71	2	0.021	0.083	0.014	0.180
natural	2	7	6	5.25	2	0.072	0.059	0.046	0.655
open	2	2	2	0.00	2	1.000	1.000	1.000	1.000
pensive	1	2	2	0.50	2	0.779	0.564	0.564	1.000
restless	8	2	3	8.86	2	0.012	0.014	0.025	0.564
sad	2	2	2	0.00	2	1.000	1.000	1.000	-
unlikable	4	4	5	0.20	2	0.905	1.000	0.705	0.739
unnatural	6	3	1	5.43	2	0.066	0.180	0.025	0.317

Frequency of mention: Number of participants mentioning at least one attribute referring to the respective semantic category in at least one of the corresponding conditions (shift down-/side-/upward).

Table S5: Analyses of shift direction in Experiment 1

Shift direction	Frequency of mention			Cochran's Q test			*p*-values of pairwise McNemar tests		
**Neutral context**	d	s	u	X²	df	*p*	d vs. s	d vs. u	s vs. u
amused	0	0	0	-	2	-	-	-	-
annoyed	0	0	1	2.00	2	0.368	-	0.317	0.317
anxious	2	0	0	4.00	2	0.135	0.157	0.157	-
attentive	5	4	5	0.20	2	0.905	0.705	1.000	0.655
avoidant	6	6	3	2.25	2	0.325	1.000	0.180	0.180
bewildered	1	4	3	4.67	2	0.097	0.083	0.157	0.317
calm	5	2	2	3.00	2	0.223	0.180	0.180	1.000
disinterested	9	6	8	1.40	2	0.497	0.180	0.705	0.480
empathetic	1	1	1	0.00	2	1.000	1.000	1.000	1.000
inattentive	6	10	8	3.43	2	0.180	0.046	0.414	0.317
indifferent	2	1	1	2.00	2	0.368	0.317	0.317	-
interested	9	6	6	2.57	2	0.276	0.180	0.180	1.000
likable	4	1	2	2.33	2	0.311	0.180	0.317	0.564
natural	4	2	2	1.60	2	0.449	0.317	0.317	1.000
open	2	5	0	7.60	2	0.022	0.083	0.157	0.025
pensive	3	3	3	0.00	2	1.000	1.000	1.000	1.000
restless	5	4	3	0.86	2	0.651	0.705	0.157	0.655
sad	1	1	1	0.00	2	1.000	1.000	1.000	-
unlikable	2	4	3	1.50	2	0.472	0.317	0.564	0.317
unnatural	2	5	3	2.80	2	0.247	0.180	0.564	0.157
**Negative context**	d	s	u	X²	df	*p*	d vs. s	d vs. u	s vs. u
amused	1	0	0	2.00	2	0.368	0.317	0.317	-
annoyed	0	0	0	-	2	-	-	-	-
anxious	1	3	1	2.00	2	0.368	0.317	-	0.317
attentive	6	6	8	1.00	2	0.607	1.000	0.414	0.317
avoidant	8	13	9	2.91	2	0.234	0.102	1.000	0.157
bewildered	3	3	1	1.60	2	0.449	1.000	0.317	0.317
calm	2	3	3	0.67	2	0.717	0.317	0.564	1.000
disinterested	6	6	14	8.91	2	0.012	1.000	0.020	0.020
empathetic	6	8	5	1.75	2	0.417	0.655	0.317	0.257
inattentive	8	9	9	0.00	2	1.000	1.000	1.000	1.000
indifferent	3	2	2	2.00	2	0.368	0.564	0.157	0.317
interested	8	7	7	0.73	2	0.695	0.480	0.414	1.000
likable	3	4	3	0.33	2	0.846	0.564	1.000	0.655
natural	7	3	2	4.67	2	0.097	0.102	0.059	0.655
open	4	3	1	3.50	2	0.174	0.564	0.083	0.157
pensive	1	2	2	0.50	2	0.779	0.564	0.564	1.000
restless	4	4	3	0.25	2	0.882	1.000	0.705	0.655
sad	3	1	2	3.00	2	0.223	0.157	0.317	0.317
unlikable	3	5	3	0.80	2	0.670	0.480	1.000	0.414
unnatural	6	2	4	4.00	2	0.135	0.046	0.317	0.317

Frequency of mention: Number of participants mentioning at least one attribute referring to the respective semantic category in at least one of the corresponding conditions (shift 2s/4s/6s). d: downward, s: sideward, u: upward.

Figure S1: Relative frequency of mention for blink frequency and shift frequency

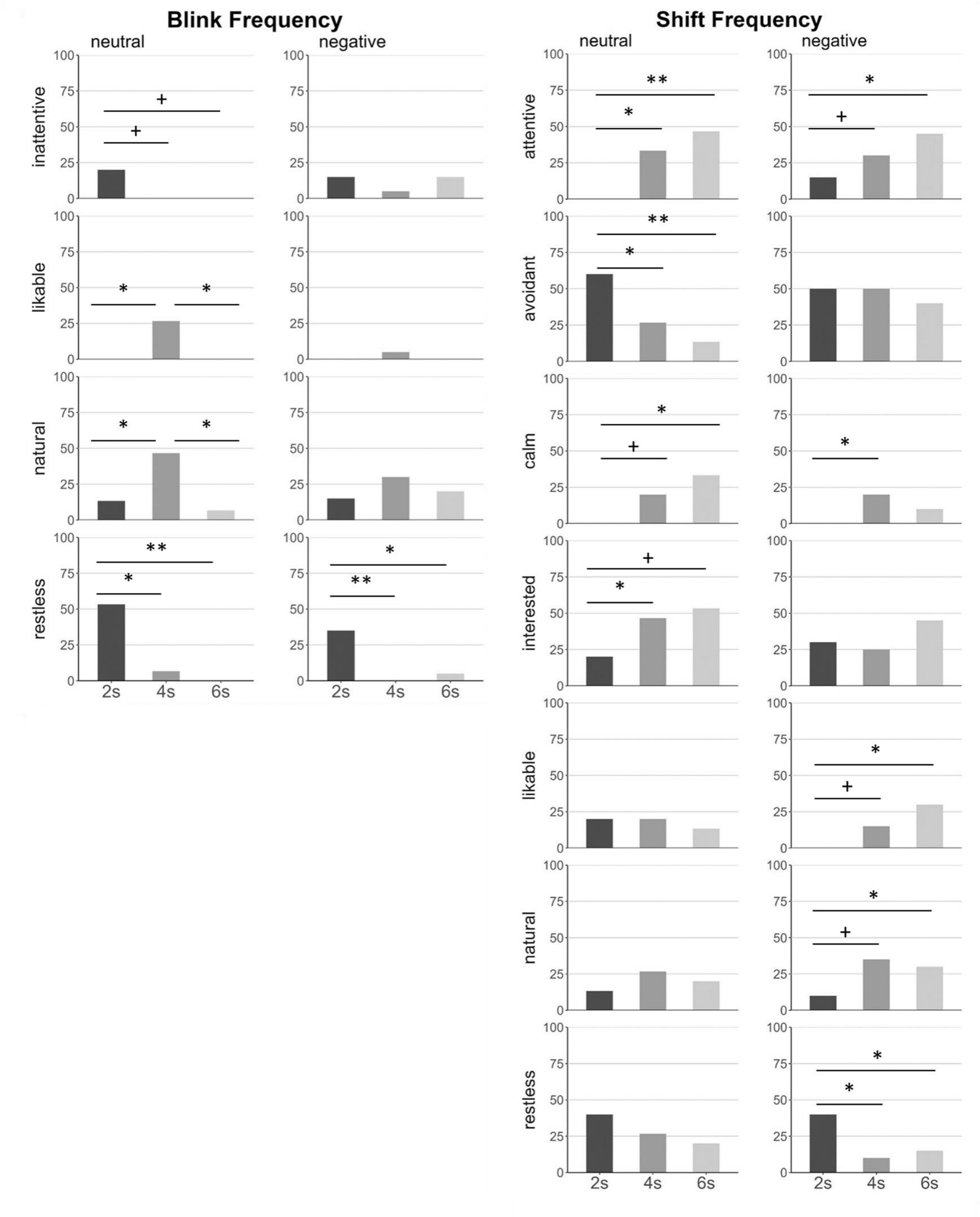

Relative frequency (in %) of participants (Exp. 1) mentioning attributes belonging to a semantic category as a function of blink frequency (left side) and shift frequency (right side). Categories for which effects met the criterion of significance are presented in alphabetical order. Dark gray columns: high frequency, medium gray columns: medium frequency, light gray columns: slow frequency. Horizontal bars indicate significant pairwise comparisons: +: $p < .10$, *: $p < .05$, **: $p < .01$, ***: $p < .001$.

Figure S2: Relative frequency of mention for constant gaze direction and shift direction

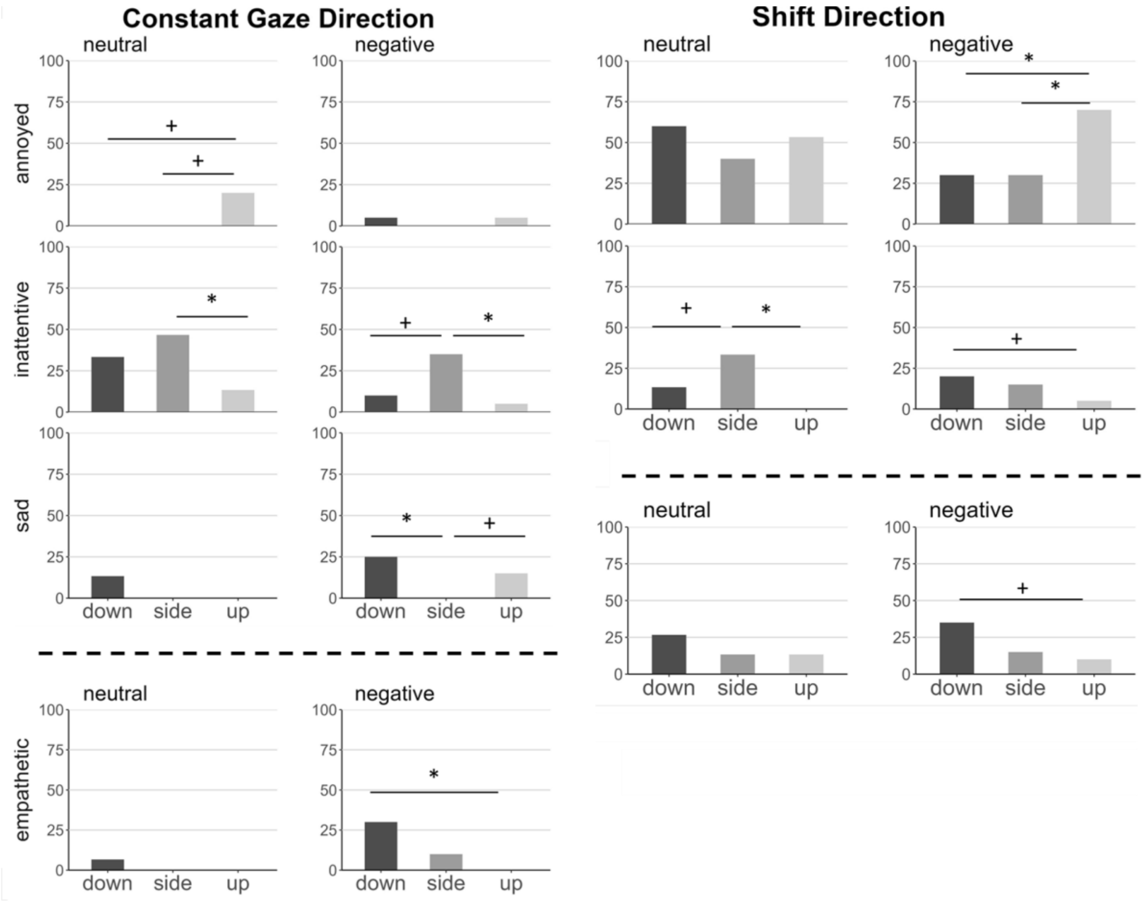

Relative frequency (in %) of participants (Exp. 1) mentioning attributes belonging to a semantic category as a function of constant gaze direction (left side) and shift direction (right side). Categories for which effects met the criterion of significance are presented in alphabetical order. Below the dotted line, the figure presents effects that did not reach this criterion but were descriptively interesting. Dark gray columns: downward gaze, medium gray columns: sideward gaze, light gray columns: upward gaze. Horizontal bars indicate significant pairwise comparisons: +: $p < .10$, *: $p < .05$, **: $p < .01$, ***: $p < .001$.

S3 Supplementary results for Experiment 2

S3.1 Self-reported motivation and diligence of participants

At the end of Experiment 2, participants were asked to indicate their level of motivation and diligence in completing the study on a Likert scale (range 1-7). Ratings in the sample ranged from 4 to 7, with a mean of 6.3 (*SD*=0.7).

S3.2 Main effects of emotional valence and interactions between emotional valence and gaze behavior

Blink frequency

The main effect of the between-subject factor emotional valence reached significance for the category 'sad' (F(1,78)=24.03, p<.001, η_p^2=.24), which was rated higher in the negative compared to the neutral condition. The main effect of emotional valence did not reach significance for any of the other categories (all Fs<3.48, all ps>.065).

The interaction between emotional valence and blink frequency reached significance for 'sad' (*F*(2,156)=3.21, *p*=.049, η_p^2=.04). One-factorial ANOVAs conducted separately for the emotional valence groups revealed that the main effect of blink frequency was not significant in the neutral group (*F*(2,80)=1.51, *p*=.229, η_p^2=.04), but in the negative group (*F*(2,76)=3.77, *p*=.031, η_p^2=.09) with significantly lower ratings for slow compared to medium-fast blinking (*t*(38)=2.91, *p*=.018, *d*=0.47, for all other pairwise comparisons *t*s<|2.1|, *p*s>.152).

Levene tests indicated that there was homogeneity of variance between the valence groups in the fast (*F*(1,78)=1.46, *p*=.231) and medium blink condition (*F*(1,78)=2.63, *p*=.109), but not in the slow blink condition (*F*(1,78)=4.00, *p*=.049).

No other interaction reached significance (all *F*s<2.49, all *p*s>.097).

Constant gaze direction

The main effect of emotional valence reached significance for 'attentive' (*F*(1,78)=6.25, *p*=.015, η_p^2=.07) and 'empathetic' (*F*(1,78)=5.48, *p*=.022, η_p^2=.07) which were both rated higher in the

negative compared to the neutral condition. The main effect of emotional valence did not reach significance for any of the other categories (all *F*s<3.40, all *p*s>.069).

The interaction between emotional valence and constant gaze direction reached significance for the category 'calm' (*F*(2,156)=5.16, *p*=.007, η_p^2=.06). One-factorial ANOVAs conducted separately for the emotional valence groups revealed that the main effect of gaze direction was not significant in the negative group (*F*(2,76)=1.00, *p*=.372, η_p^2=.03), but in the neutral group (*F*(2,80)=4.76, *p*=.013, η_p^2=.11) with significantly higher ratings in the downward compared to the sideward gaze condition (*t*(40)=3.50, *p*=.003, *d*=0.55, for all other pairwise comparisons *t*s<|1.98|, *p*s>.160). According to Levene tests, homogeneity of variance between emotional valence groups was present in all conditions of gaze direction (all *F*s<3.25, all *p*>.075).

The interaction also reached significance for 'empathetic' (*F*(2,156)=6.05, *p*=.003, η_p^2=.07). One-factorial ANOVAs conducted separately for the emotional valence groups revealed that the main effect of gaze direction was not significant in the neutral group (*F*(2,80)=2.55, *p*=.087, η_p^2=.06), but in the negative group (*F*(2,76)=18.54, *p*<.001, η_p^2=.33) with significantly lower ratings for downward compared to sideward (*t*(38)=5.30, *p*<.001, *d*=0.85) and upward (*t*(38)=4.75, *p*<.001, *d*=0.76) gaze (sideward vs. upward gaze: *t*<|1|). Levene tests indicated that there was homogeneity of variance between the emotional valence groups in the sideward (*F*(1,78)=1.20, *p*=.277) and upward gaze condition (*F*(1,78)<1), but not in the downward gaze condition (*F*(1,78)=4.60, *p*=.035).

No other interaction reached significance (all *F*s<2.81, all *p*s>.072).

Gaze shifts

The main effect of emotional valence reached significance for 'attentive' (*F*(1,78)=4.64, *p*=.034, η_p^2=.06) and 'sad' (*F*(1,78)=6.95, *p*=.010, η_p^2=.08) which were rated higher in the negative compared to the neutral condition, as well as for 'open' (*F*(1,78)=5.51, *p*=.021, η_p^2=.07) which was rated higher

in the neutral condition. The main effect of emotional valence did not reach significance for any of the other categories (all *F*s<3.41, all *p*s>.068).

Shift frequency

There was a significant interaction between emotional valence and shift frequency for ‘natural’ ($F(2,156)=3.40$, $p=.048$, $\eta_p^2=.04$). Two-factorial ANOVAs (with the factors shift frequency and shift direction) conducted separately for the emotional valence groups revealed that the main effect of shift frequency reached significance in both the neutral ($F(2,80)=33.99$, $p<.001$, $\eta_p^2=.46$) and the negative group ($F(1,76)=8.66$, $p=.001$, $\eta_p^2=.19$), with ratings decreasing with increasing shift frequency. However, the effect was more pronounced in the neutral group, in which all pairwise comparisons reached significance (2s vs. 4s: $t(40)=-5.23$, $p<.001$, $d=-0.82$; 2s vs. 6s: $t(40)=-6.67$, $p<.001$, $d=-1.04$; 4s vs. 6s: $t(40)=-4.05$, $p=.001$, $d=-0.63$) while in the negative group only the comparison between slow and fast shifts did (2s vs. 4s: $t(38)=-2.35$, $p=.072$, $d=-0.38$; 2s vs. 6s: $t(38)=-3.51$, $p=.004$, $d=-0.56$; 4s vs. 6s: $t(38)=-2.33$, $p=.075$, $d=-0.37$). According to Levene tests, homogeneity of variance between emotional valence groups was present in all conditions of shift frequency (all *F*s<1).

The interaction also reached significance for ‘sad’ ($F(2,156)=6.99$, $p=.001$, $\eta_p^2=-.08$. Two-factorial ANOVAs conducted separately for the emotional valence groups revealed no significant main effect of shift frequency in the neutral group ($F(2,80)=2.78$, $p=.069$, $\eta_p^2=.07$), but in the negative group ($F(2,76)=4.72$, $p=.012$, $\eta_p^2=.11$), in which ratings were higher in the slow shift compared to the fast shift condition ($t(38)=-2.95$, $p=.016$, $d=-0.47$; all other pairwise comparisons: ts<|2.32|, ps>.078). According to Levene tests, homogeneity of variance between emotional valence groups was present in all conditions of shift frequency (all *F*s<1.16, all *p*s>.286).

No other interaction reached significance (all *F*s<3.09, all *p*s>.052).

Shift direction

The interaction between emotional valence and shift direction reached significance for ‘attentive’ ($F(2,156)=8.34$, $p<.001$, $\eta_p^2=.10$). Two-factorial ANOVAs conducted separately for the emotional valence groups revealed that the main effect of shift direction was significant in both groups (neutral: $F(2,80)=4.13$, $p=.020$, $\eta_p^2=.09$, negative: $F(2,76)=46.78$, $p<.001$, $\eta_p^2=.55$), but was more pronounced in the negative group, in which the downward shift condition had significantly higher ratings compared to sideward and upward shifts (down vs. side: $t(38)=7.60$, $p<.001$, $d=1.22$; down vs. up: $t(38)=8.20$, $p<.001$, $d=1.31$; side vs. up: $t(38)<1$), while only the comparison between downward and sideward shifts reached significance in the neutral group ($t(40)=2.54$, $p=.046$, $d=0.40$; all other comparisons: ts<2.31, ps$>.079$). According to Levene tests, homogeneity of variance between emotional valence groups was present in all conditions of shift direction (all Fs<1).

There was also a significant interaction for ‘bewildered’ ($F(2,156)=4.45$, $p=.013$, $\eta_p^2=.05$). Two-factorial ANOVAs conducted separately for the emotional valence groups revealed that the effect was only significant in the negative group (neutral: $F(2,80)=1.29$, $p=.280$, $\eta_p^2=.03$, negative: $F(2,76)=4.63$, $p=.014$, $\eta_p^2=.11$), with significantly lower ratings in the upward compared to the sideward shift condition ($t(38)=2.88$, $p=.019$, $d=0.46$, all other pairwise comparisons: ts$<|2.19|$, ps$>.104$). According to Levene tests, homogeneity of variance between emotional valence groups was present in all conditions of shift direction (all Fs<1).

The interaction between emotional valence and shift direction also reached significance for ‘empathetic’ ($F(2,156)=10.36$, $p<.001$, $\eta_p^2=.12$). Two-factorial ANOVAs conducted separately for the emotional valence groups revealed that the main effect of gaze direction reached significance in both groups (neutral: $F(2,80)=3.48$, $p=.036$, $\eta_p^2=.08$; negative: $F(2,76)=32.51$, $p<.001$, $\eta_p^2=.46$). However, in the neutral group, none of the pairwise comparisons reached significance after alpha adjustment (all ts$<|2.40|$, all ps$>.064$), while in the negative group, ratings were significantly higher for the downward compared to the sideward and upward shift condition (down vs. side: $t(38)=5.71$, $p<.001$, $d=0.91$; down vs. up: $t(38)=8.14$, $p=<.001$, $d=1.30$; side vs. up: $t(38)<1$). Levene tests indicated that there was

homogeneity of variance between the emotional valence groups in the sideward (*F*(1,78)<1) and upward gaze condition (*F*(1,78)=1.49, *p*=.225), but not in the downward gaze condition (*F*(1,78)=4.38, *p*=.040).

There was a significant interaction between emotional valence and shift direction for ‘interested’ (*F*(2,156)=9.90, *p*<.001, η_p^2=.11). Two-factorial ANOVAs conducted separately for the emotional valence groups revealed that the main effect of shift direction was not significant in the neutral group (*F*(2,80)=2.68, *p*=.078, η_p^2=.06), but in the negative group (*F*(2,76)=38.52, *p*<.001, η_p^2=.50), with significantly higher ratings in the downward compared to the sideward and upward shift condition (down vs. side: *t*(38)=6.06, *p*<.001, *d*=0.97; down vs. up: *t*(38)=9.04, *p*<.001, *d*=1.45; side vs. up: *t*(38)=1.29, *p*=.615, *d*=0.21). According to Levene tests, homogeneity of variance between emotional valence groups was present in all conditions of shift direction (all *F*s<1).

The interaction between emotional valence and shift direction reached significance for ‘likable’ (*F*(2,156)=5.35, *p*=.006, η_p^2=.06). Two-factorial ANOVAs conducted separately for the emotional valence groups revealed that the main effect of shift direction was significant in both emotional valence groups (neutral: *F*(2,80)=7.56, *p*=.001, η_p^2=.16; negative: *F*(2,76)=36.54, *p*<.001, η_p^2=.49). Downward shifts were rated significantly higher compared to sideward and upward shifts in both the neutral (down vs. side: *t*(40)=3.40, *p*=.005, *d*=0.53; down vs. up: *t*(40)=3.31, *p*=.006, *d*=0.52; side vs. up: *t*(40)<1) and the negative group (down vs. side: *t*(38)=5.24, *p*<.001, *d*=0.84; down vs. up: *t*(38)=8.83, *p*<.001, *d*=1.41; side vs. up: *t*(38)=2.31, *p*=.079, *d*=0.37), but the differences were more pronounced in the negative group. According to Levene tests, homogeneity of variance between emotional valence groups was present in all conditions of shift direction (all *F*s<1).

There was a significant interaction between emotional valence and shift direction for ‘sad’ (*F*(2,156)=8.04, *p*=.001, η_p^2=.09). Two-factorial ANOVAs conducted separately for the emotional valence groups revealed that the main effect of shift direction was significant both in the neutral (*F*(2,80)=8.61, *p*=.001, η_p^2=.18) and the negative group (*F*(2,76)=50.39, *p*<.001, η_p^2=.57). In the

negative group downward gaze was rated significantly higher compared to sideward and upward gaze (down vs. side: $t(38)=9.04$, $p<.001$, $d=1.45$; down vs. up: $t(38)=7.97$, $p<.001$, $d=1.28$; side vs. up: $t(38)<1$), while in the neutral group only the comparison between downward and upward gaze reached significance (down vs. side: $t(40)=2.27$, $p=.086$, $d=0.35$; down vs. up: $t(40)=4.07$, $p=.001$, $d=0.64$; side vs. up: $t(40)=1.79$, $p=.242$, $d=0.28$). According to Levene tests, homogeneity of variance between emotional valence groups was present in all conditions of shift direction (all *F*s<1).
No other interaction reached significance (all *F*s<2.65, all *p*s>.077).

S3.3 Interactions between shift direction and shift frequency (exploratory analyses)

The interaction between shift direction and shift frequency reached significance for the attribute 'attentive' ($F(4,312)=3.16$, $p=.016$, $\eta_p^2=.04$). To explore this effect, we conducted one-factorial ANOVAs with the within factor shift frequency separately for the gaze direction conditions. The main effect of shift frequency was significant for all three gaze directions (down: $F(2,158)=8.42$, $p<.001$, $\eta_p^2=.10$; side: $F(2,158)=25.67$, $p<.001$, $\eta_p^2=.25$; up: $F(2,158)=26.72$, $p<.001$, $\eta_p^2=.25$). Ratings decreased with increasing shift frequency in all conditions, with higher effect sizes in the sideward and upward shift compared to the downward shift condition. In the upward shift condition, all pairwise comparisons reached significance (all *t*s>|3.67|, all *p*s<.001, all *d*s>|0.41|), while only the comparisons between fast and slow and medium-fast and slow shifts did in the downward (2s vs. 4s: $t(79)<|1|$; 2s vs. 6s: $t(79)=-3.77$, $p=.001$ $d=-0.42$; 4s vs. 6s: $t(79)=-3.19$, $p=.006$, $d=-0.36$) and sideward shift condition (2s vs. 4s: $t(79)=-1.30$, $p=.597$, $d=-0.14$; 2s vs. 6s: $t(79)=-6.28$, $p<.001$ $d=-0.70$; 4s vs. 6s: $t(79)=-6.09$, $p<.001$, $d=-.0.68$).
There was also a significant interaction for 'empathetic' ($F(4,312)=3.23$, $p=.016$, $\eta_p^2=.04$). One-factorial ANOVAs conducted separately for gaze directions showed that there was no significant effect of gaze frequency in the conditions with downward shifts ($F(2,158)=1.65$, $p=.197$, $\eta_p^2=.02$). In the sideward ($F(2,158)=18.21$, $p<.001$, $\eta_p^2=.19$) and upward shift conditions ($F(2,158)=18.51$, $p<.001$,

η_p^2=.19), slow shifts were rated significantly higher than the other two shift frequencies (all *t*s>|4.13|, all *p*s<.001, all *d*>|0.46|), with no significant difference between fast and medium-fast shifts (all *t*s<|2.17|, all *p*s>.101).

The interaction for 'natural' also reached significance (*F*(4,312)=3.46, *p*=.010, η_p^2=.04). One-factorial ANOVAs conducted separately for shift directions revealed significant main effects of shift frequency for all shift directions (down: *F*(2,158)=4.75, *p*=.013, η_p^2=.06; side: *F*(2,158)=14.04, *p*<.001, η_p^2=.15; up: *F*(2,158)=29.23, *p*<.001, η_p^2=.27), with higher ratings corresponding to lower shift frequencies. However, for downward shifts, only the comparison between fast and medium-fast shifts reached significance (*t*(79)=-2.61, *p*=.032, *d*=-0.29; for all other comparisons: *t*s<|2.42|, *p*s>.054)., while for sideward shifts both the difference between fast and medium-fast and fast and slow reached significance (2s vs. 4s: *t*(79)=-3.12, *p*=.007, *d*=-0.35; 2s vs. 6s: *t*(79)=-4.98, *p*<.001, *d*=-0.56; 4s vs. 6s: *t*(79)=-2.27, *p*=.078, *d*=-0.25) and for upward shifts all pairwise comparisons were significant (all *t*s>|3.88|, all *p*s<.001, all *d*s>|0.43|). No further interactions between shift direction and shift frequency reached significance (all *F*s<2.21, all *p*s>.075).

To sum up the exploratory analyses, it seems that the influence of shift frequency on the perception of specific attributes was less pronounced when the gaze was shifted downward, as opposed to when it was shifted upwards or to the side.

Table S6: Main effects of gaze behavior in Experiment 2

	M			*SD*			Main Effect Gaze Behavior						
**Blink frequency**	2s	4s	6s	2s	4s	6s	ε	df1	df2	*F*	*p*	p_{adj}	η_p^2
annoyed	2.9	2.2	2.1	1.5	1.2	1.2	0.898	2	154	11.23	0.000	0.001	0.13
attentive	4.7	5.3	5.4	1.4	1.3	1.3	0.820	2	156	11.35	0.000	0.001	0.13
bewildered	2.8	2.1	2.3	1.5	1.2	1.4	0.923	2	156	9.76	0.000	0.002	0.11
calm	3.9	5.4	5.7	1.6	1.3	1.1	0.895	2	156	49.97	0.000	0.000	0.39
empathetic	3.7	3.9	4.0	1.4	1.7	1.6	0.947	2	156	1.71	0.186	1.000	0.02
interested	4.2	4.6	4.7	1.5	1.5	1.5	0.877	2	156	5.40	0.008	0.077[a]	0.06
likeable	4.2	4.5	4.5	1.3	1.4	1.5	0.984	2	156	2.75	0.068	0.678	0.03
natural	3.7	4.7	4.5	1.6	1.6	1.7	0.878	2	156	12.18	0.000	0.000	0.14
open	4.3	4.7	5.0	1.4	1.3	1.4	0.915	2	156	9.97	0.000	0.001	0.11
sad	2.4	2.5	2.2	1.4	1.4	1.3	0.891	2	156	2.57	0.087	0.867	0.03
**Constant gaze direction**	d	s	u	d	s	u	ε	df1	df2	*F*	*p*	p_{adj}	η_p^2
annoyed	2.9	4.1	4.3	1.5	1.8	2.1	0.916	2	156	19.10	0.000	0.000	0.20
attentive	3.4	2.1	3.2	1.7	1.2	1.9	0.898	2	156	17.18	0.000	0.000	0.18
bewildered	2.9	2.9	2.8	1.6	1.6	1.8	0.946	2	156	0.34	0.698	1.000	0.00
calm	5.1	4.8	4.9	1.6	1.6	1.6	0.964	2	156	0.88	0.414	1.000	0.01
empathetic	3.0	1.9	2.2	1.8	1.1	1.5	0.998	2	156	17.90	0.000	0.000	0.19
interested	3.0	1.9	2.6	1.6	1.1	1.8	0.937	2	156	14.89	0.000	0.000	0.16
likeable	3.5	2.5	2.7	1.5	1.2	1.4	0.975	2	156	16.51	0.000	0.000	0.17
natural	3.6	2.9	2.7	1.5	1.4	1.5	0.944	2	156	10.37	0.000	0.001	0.12
open	2.9	1.8	2.6	1.5	1.0	1.7	0.851	2	156	16.18	0.000	0.000	0.17
sad	3.7	2.6	2.2	1.9	1.5	1.4	0.766	2	156	33.70	0.000	0.000	0.30
**Shift frequency**	2s	4s	6s	2s	4s	6s	ε	df1	df2	*F*	*p*	p_{adj}	η_p^2
annoyed	3.8	3.5	2.9	1.2	1.1	1.1	0.958	2	156	30.83	0.000	0.000	0.28
attentive	3.0	3.4	4.1	1.1	0.9	1.0	0.912	2	156	39.02	0.000	0.000	0.33
bewildered	3.1	2.7	2.3	1.3	1.1	1.1	0.937	2	156	27.36	0.000	0.000	0.26
calm	2.9	4.0	4.8	1.0	1.0	1.0	0.864	2	156	104.83	0.000	0.000	0.57
empathetic	2.8	3.1	3.5	1.0	0.8	1.1	0.925	2	156	24.94	0.000	0.000	0.24
interested	2.8	3.0	3.7	1.0	0.7	1.0	0.894	2	156	37.10	0.000	0.000	0.32
likeable	3.3	3.5	3.9	0.9	0.8	1.0	0.930	2	156	24.81	0.000	0.000	0.24
natural	3.4	3.9	4.2	1.1	1.0	1.2	0.779	2	156	37.64	0.000	0.000	0.33
open	2.7	3.0	3.7	0.8	0.8	0.9	0.933	2	156	44.21	0.000	0.000	0.36
sad	2.5	2.6	2.6	1.2	1.1	1.2	0.993	2	156	1.12	0.329	1.000	0.01
**Shift direction**	d	s	u	d	s	u	ε	df1	df2	*F*	*p*	p_{adj}	η_p^2
annoyed	2.9	3.6	3.8	1.2	1.3	1.3	0.964	2	156	22.25	0.000	0.000	0.22
attentive	4.1	3.2	3.2	1.2	1.0	0.9	0.975	2	156	35.28	0.000	0.000	0.31
bewildered	2.7	2.9	2.6	1.2	1.2	1.3	0.997	2	156	1.77	0.174	1.000	0.02
calm	4.2	3.6	4.0	1.0	1.0	1.1	0.983	2	156	8.60	0.000	0.003	0.10
empathetic	3.7	2.8	2.8	1.3	1.0	1.0	0.952	2	156	30.40	0.000	0.000	0.28
interested	3.8	2.9	2.8	1.2	0.9	0.9	0.938	2	156	30.14	0.000	0.000	0.28
likeable	4.1	3.4	3.2	1.1	0.9	0.9	0.982	2	156	38.29	0.000	0.000	0.33
natural	4.3	3.8	3.4	1.2	1.2	1.0	0.992	2	156	25.47	0.000	0.000	0.25
open	3.5	2.9	2.9	1.0	0.9	0.9	0.968	2	156	15.48	0.000	0.000	0.17
sad	3.3	2.2	2.1	1.6	1.1	1.1	0.894	2	156	47.50	0.000	0.000	0.38

d: downward, s: sideward, u: upward.
The column p_{adj} contains p values after correcting for multiple analyses (10 analyses per core feature).
[a] marks the single main effect that no longer reached significance after alpha-correction.

Table S7: Pairwise *t*-tests between conditions of gaze behavior in Experiment 2

	t-test: 2s vs. 4s				*t*-test: 2s vs. 6s				*t*-test: 4s vs. 6s			
**Blink frequency**	df	*t*	*p*	*d*	df	*t*	*p*	*d*	df	*t*	*p*	*d*
annoyed	78	3.43	0.003	0.39	78	4.12	0.000	0.46	79	0.81	1.000	0.09
attentive	79	-3.50	0.002	-0.39	79	-3.74	0.001	-0.42	79	-0.69	1.000	-0.08
bewildered	79	4.08	0.000	0.46	79	2.77	0.021	0.31	79	-1.41	0.486	-0.16
calm	79	-6.92	0.000	-0.77	79	-8.91	0.000	-1.00	79	-2.40	0.057	-0.27
empathetic	79	-1.19	0.708	-0.13	79	-1.77	0.242	-0.20	79	-0.47	1.000	-0.05
interested	79	-1.93	0.172	-0.22	79	-2.94	0.013	-0.33	79	-1.31	0.585	-0.15
likeable	79	-1.72	0.269	-0.19	79	-2.16	0.102	-0.24	79	-0.40	1.000	-0.04
natural	79	-4.08	0.000	-0.46	79	-3.63	0.001	-0.41	79	0.93	1.000	0.10
open	79	-2.45	0.050	-0.27	79	-4.06	0.000	-0.45	79	-2.10	0.117	-0.23
sad	79	-0.85	1.000	-0.10	79	1.17	0.735	0.13	79	2.70	0.025	0.30

	t-test: down vs. side				*t*-test: down vs. up				*t*-test: side vs. up			
**Constant gaze direction**	df	*t*	*p*	*d*	df	*t*	*p*	*d*	df	*t*	*p*	*d*
annoyed	79	-5.71	0.000	-0.64	79	-5.02	0.000	-0.56	79	-0.64	1.000	-0.07
attentive	79	6.25	0.000	0.70	79	0.88	1.000	0.10	79	-4.53	0.000	-0.51
bewildered	79	-0.14	1.000	-0.02	79	0.61	1.000	0.07	79	0.77	1.000	0.09
calm	79	1.38	0.513	0.15	79	1.00	0.966	0.11	79	-0.25	1.000	-0.03
empathetic	79	5.47	0.000	0.61	79	3.92	0.001	0.44	79	-1.52	0.399	-0.17
interested	79	6.02	0.000	0.67	79	1.74	0.257	0.19	79	-3.43	0.003	-0.38
likeable	79	4.96	0.000	0.55	79	4.56	0.000	0.51	79	-0.86	1.000	-0.10
natural	79	3.33	0.004	0.37	79	4.00	0.000	0.45	79	1.16	0.753	0.13
open	79	6.13	0.000	0.69	79	1.12	0.801	0.13	79	-4.40	0.000	-0.49
sad	79	5.21	0.000	0.58	79	6.68	0.000	0.75	79	3.23	0.005	0.36

	t-test: 2s vs. 4s				*t*-test: 2s vs. 6s				*t*-test: 4s vs. 6s			
**Shift frequency**	df	*t*	*p*	*d*	df	*t*	*p*	*d*	df	*t*	*p*	*d*
annoyed	79	3.15	0.007	0.35	79	7.37	0.000	0.82	79	5.19	0.000	0.58
attentive	79	-2.82	0.018	-0.32	79	-7.76	0.000	-0.87	79	-6.77	0.000	-0.76
bewildered	79	3.98	0.000	0.45	79	6.61	0.000	0.74	79	4.00	0.000	0.45
calm	79	-10.33	0.000	-1.15	79	-12.62	0.000	-1.41	79	-6.12	0.000	-0.68
empathetic	79	-2.66	0.028	-0.30	79	-6.19	0.000	-0.69	79	-4.81	0.000	-0.54
interested	79	-2.77	0.021	-0.31	79	-7.28	0.000	-0.81	79	-6.49	0.000	-0.73
likeable	79	-2.16	0.101	-0.24	79	-6.11	0.000	-0.68	79	-5.33	0.000	-0.60
natural	79	-5.24	0.000	-0.59	79	-7.06	0.000	-0.79	79	-4.49	0.000	-0.50
open	79	-3.26	0.005	-0.36	79	-8.26	0.000	-0.92	79	-6.30	0.000	-0.70
sad	79	-1.02	0.933	-0.11	79	-1.33	0.558	-0.15	79	-0.30	1.000	-0.03

	t-test: down vs. side				*t*-test: down vs. up				*t*-test: side vs. up			
**Shift direction**	df	*t*	*p*	*d*	df	*t*	*p*	*d*	df	*t*	*p*	*d*
annoyed	79	-4.95	0.000	-0.55	79	-5.82	0.000	-0.65	79	-1.79	0.231	-0.20
attentive	79	6.49	0.000	0.73	79	6.52	0.000	0.73	79	0.32	1.000	0.04
bewildered	79	-1.42	0.480	-0.16	79	0.30	1.000	0.03	79	1.62	0.327	0.18
calm	79	4.23	0.000	0.47	79	1.35	0.540	0.15	79	-2.53	0.040	-0.28

empathetic	79	5.64	0.000	0.63	79	6.40	0.000	0.72	79	0.19	1.000	0.02
interested	79	5.14	0.000	0.58	79	6.70	0.000	0.75	79	1.10	0.819	0.12
likeable	79	6.09	0.000	0.68	79	7.69	0.000	0.86	79	1.80	0.227	0.20
natural	79	3.31	0.004	0.37	79	7.36	0.000	0.82	79	3.68	0.001	0.41
open	79	4.62	0.000	0.52	79	4.45	0.000	0.50	79	-0.45	1.000	-0.05
sad	79	6.56	0.000	0.73	79	8.11	0.000	0.91	79	1.24	0.657	0.14

p-values for *t*-tests exploring the same main effect are Bonferroni-adjusted.

Table S8: Main effects of emotional valence in Experiment 2

	M		*SD*		Main Effect Valence				
**Blink frequency**	Neut	Neg	Neut	Neg	df1	df2	*F*	*p*	η_p^2
annoyed	2.4	2.4	0.9	0.9	1	77	0.00	0.963	0.00
attentive	5.3	5.0	0.9	1.0	1	78	1.36	0.246	0.02
bewildered	2.5	2.3	1.0	1.0	1	78	0.67	0.417	0.01
calm	5.0	4.9	0.9	0.9	1	78	0.38	0.537	0.00
empathetic	4.0	3.8	1.3	1.3	1	78	0.47	0.497	0.01
interested	4.6	4.4	1.2	1.2	1	78	0.97	0.328	0.01
likeable	4.6	4.2	1.0	1.1	1	78	3.26	0.075	0.04
natural	4.4	4.1	1.2	1.2	1	78	1.50	0.224	0.02
open	4.9	4.4	0.9	1.1	1	78	3.48	0.066	0.04
sad	1.8	2.9	0.9	1.1	1	78	24.03	0.000	0.24
**Constant gaze direction**	Neut	Neg	Neut	Neg	df1	df2	*F*	*p*	η_p^2
annoyed	4.0	3.6	1.4	1.2	1	78	1.80	0.184	0.02
attentive	2.6	3.2	1.0	1.1	1	78	6.25	0.015	0.07
bewildered	3.0	2.7	1.2	1.2	1	78	1.11	0.296	0.01
calm	4.8	5.0	1.2	1.3	1	78	0.44	0.507	0.01
empathetic	2.1	2.6	0.9	1.2	1	78	5.48	0.022	0.07
interested	2.3	2.7	1.0	1.1	1	78	3.40	0.069	0.04
likeable	2.9	2.9	1.0	1.0	1	78	0.00	0.953	0.00
natural	2.9	3.2	1.0	1.0	1	78	2.52	0.116	0.03
open	2.3	2.5	1.1	0.9	1	78	0.60	0.441	0.01
sad	2.8	2.8	1.3	1.3	1	78	0.01	0.906	0.00
**Gaze shifts**	Neut	Neg	Neut	Neg	df1	df2	*F*	*p*	η_p^2
annoyed	3.5	3.3	0.9	1.1	1	78	0.53	0.471	0.01
attentive	3.3	3.7	0.7	0.8	1	78	4.64	0.034	0.06
bewildered	2.9	2.6	1.1	1.0	1	78	1.80	0.183	0.02
calm	3.8	4.0	0.6	0.9	1	78	0.96	0.331	0.01
empathetic	3.0	3.3	0.7	0.9	1	78	3.00	0.087	0.04
interested	3.0	3.3	0.7	0.7	1	78	2.48	0.119	0.03
likeable	3.7	3.4	0.7	0.7	1	78	3.41	0.068	0.04
natural	3.8	3.9	1.0	0.9	1	78	0.57	0.454	0.01
open	3.3	2.9	0.7	0.6	1	78	5.51	0.021	0.07
sad	2.3	2.8	1.0	1.0	1	78	6.95	0.010	0.08

Table S9: Interactions between emotional valence and gaze behavior in Experiment 2

	Interaction Valence x Gaze Behavior					
**Blink frequency**	ε	df1	df2	*F*	*p*	η_p^2
annoyed	0.898	2	154	0.74	0.467	0.01
attentive	0.820	2	156	2.49	0.098	0.03
bewildered	0.923	2	156	1.23	0.293	0.02
calm	0.895	2	156	0.21	0.784	0.00
empathetic	0.947	2	156	1.34	0.264	0.02
interested	0.877	2	156	1.78	0.177	0.02
likeable	0.984	2	156	0.60	0.547	0.01
natural	0.878	2	156	0.81	0.432	0.01
open	0.915	2	156	1.79	0.173	0.02
sad	0.891	2	156	3.21	0.049	0.04
**Constant gaze direction**	ε	df1	df2	*F*	*p*	η_p^2
annoyed	0.916	2	156	0.68	0.494	0.01
attentive	0.898	2	156	2.47	0.094	0.03
bewildered	0.946	2	156	1.38	0.255	0.02
calm	0.964	2	156	5.16	0.007	0.06
empathetic	0.998	2	156	6.05	0.003	0.07
interested	0.937	2	156	1.79	0.173	0.02
likeable	0.975	2	156	0.97	0.381	0.01
natural	0.944	2	156	0.04	0.955	0.00
open	0.851	2	156	2.81	0.072	0.03
sad	0.766	2	156	2.69	0.086	0.03
**Shift frequency**	ε	df1	df2	*F*	*p*	η_p^2
annoyed	0.958	2	156	0.24	0.776	0.00
attentive	0.912	2	156	2.92	0.062	0.04
bewildered	0.937	2	156	0.94	0.387	0.01
calm	0.864	2	156	1.56	0.217	0.02
empathetic	0.925	2	156	0.14	0.856	0.00
interested	0.894	2	156	0.41	0.640	0.01
likeable	0.930	2	156	0.51	0.589	0.01
natural	0.779	2	156	3.40	0.048	0.04
open	0.933	2	156	3.08	0.052	0.04
sad	0.993	2	156	6.99	0.001	0.08
**Shift direction**	ε	df1	df2	*F*	*p*	η_p^2
annoyed	0.964	2	156	2.64	0.077	0.03
attentive	0.975	2	156	8.34	0.000	0.10
bewildered	0.997	2	156	4.45	0.013	0.05
calm	0.983	2	156	1.45	0.238	0.02
empathetic	0.952	2	156	10.36	0.000	0.12
interested	0.938	2	156	9.90	0.000	0.11
likeable	0.982	2	156	5.35	0.006	0.06
natural	0.992	2	156	1.07	0.346	0.01
open	0.968	2	156	1.87	0.158	0.02
sad	0.894	2	156	8.04	0.001	0.09

Table S10: Main effects of gaze behavior in the neutral context group in Experiment 2

**Neutral**	*M*			*SD*			Main Effect Gaze Behavior					
**Blink frequency**	2s	4s	6s	2s	4s	6s	ε	df1	df2	*F*	*p*	η_p^2
annoyed	2.8	2.2	2.1	1.6	1.2	1.2	0.914	2	78	3.55	0.038	0.08
attentive	5.0	5.5	5.4	1.3	1.0	1.4	0.738	2	80	2.32	0.121	0.05
bewildered	2.7	2.2	2.4	1.5	1.3	1.3	0.937	2	80	2.02	0.142	0.05
calm	3.9	5.4	5.8	1.5	1.2	1.0	0.877	2	80	35.47	0.000	0.47
empathetic	3.9	3.9	4.1	1.3	1.8	1.7	0.955	2	80	0.44	0.634	0.01
interested	4.5	4.7	4.7	1.3	1.5	1.6	0.755	2	80	0.62	0.497	0.02
likeable	4.5	4.6	4.7	1.2	1.3	1.4	0.991	2	80	0.72	0.490	0.02
natural	4.0	4.7	4.6	1.6	1.7	1.6	0.894	2	80	3.56	0.038	0.08
open	4.7	4.8	5.1	1.2	1.1	1.1	0.922	2	80	2.75	0.075	0.06
sad	2.0	1.8	1.7	1.2	1.1	1.1	0.789	2	80	1.51	0.229	0.04
**Constant gaze direction**	d	s	u	d	s	u	ε	df1	df2	*F*	*p*	η_p^2
annoyed	3.0	4.5	4.4	1.6	1.7	2.2	0.834	2	80	12.70	0.000	0.24
attentive	2.8	2.0	3.0	1.6	1.1	1.8	0.937	2	80	5.69	0.006	0.12
bewildered	2.9	3.3	2.9	1.4	1.6	1.7	0.846	2	80	1.45	0.241	0.04
calm	5.3	4.5	4.7	1.5	1.7	1.5	0.934	2	80	4.76	0.013	0.11
empathetic	2.3	1.8	2.1	1.5	0.9	1.4	0.965	2	80	2.55	0.087	0.06
interested	2.6	1.8	2.5	1.5	0.9	1.6	0.878	2	80	5.87	0.006	0.13
likeable	3.4	2.5	2.8	1.5	1.2	1.4	0.958	2	80	5.77	0.005	0.13
natural	3.5	2.7	2.5	1.5	1.3	1.4	0.929	2	80	6.90	0.002	0.15
open	2.5	1.9	2.6	1.5	1.1	1.6	0.879	2	80	4.87	0.013	0.11
sad	3.5	2.8	2.2	2.1	1.5	1.4	0.733	2	80	9.07	0.001	0.18
**Shift frequency**	2s	4s	6s	2s	4s	6s	ε	df1	df2	*F*	*p*	η_p^2
annoyed	4.0	3.5	3.0	1.2	1.0	1.0	0.962	2	80	17.46	0.000	0.30
attentive	2.7	3.3	3.9	1.0	0.8	1.0	0.786	2	80	35.15	0.000	0.47
bewildered	3.3	2.8	2.4	1.3	1.2	1.1	0.854	2	80	17.88	0.000	0.31
calm	2.8	3.9	4.8	1.0	0.8	0.9	0.803	2	80	69.69	0.000	0.64
empathetic	2.6	2.9	3.4	0.8	0.7	1.0	0.792	2	80	13.91	0.000	0.26
interested	2.6	3.0	3.6	0.9	0.7	1.0	0.703	2	80	21.32	0.000	0.35
likeable	3.3	3.6	4.1	0.9	0.8	0.9	0.835	2	80	17.81	0.000	0.31
natural	3.1	3.9	4.3	1.0	1.0	1.2	0.758	2	80	33.99	0.000	0.46
open	2.7	3.3	3.7	0.9	0.7	0.9	0.838	2	80	26.71	0.000	0.40
sad	2.3	2.4	2.1	1.1	1.2	1.0	0.977	2	80	2.78	0.069	0.07
**Shift direction**	d	s	u	d	s	u	ε	df1	df2	*F*	*p*	η_p^2
annoyed	3.1	3.5	3.8	1.2	1.0	1.2	0.966	2	80	5.57	0.006	0.12
attentive	3.6	3.1	3.2	1.1	1.0	1.0	0.988	2	80	4.13	0.020	0.09
bewildered	2.7	2.9	3.0	1.2	1.2	1.3	0.985	2	80	1.29	0.280	0.03
calm	4.0	3.7	3.8	0.9	0.8	0.9	0.946	2	80	3.06	0.056	0.07
empathetic	3.2	2.8	2.9	1.0	1.0	1.0	0.987	2	80	3.48	0.036	0.08
interested	3.3	2.9	2.9	1.1	1.0	0.9	0.946	2	80	2.68	0.078	0.06
likeable	4.0	3.5	3.5	1.0	0.9	0.9	0.967	2	80	7.56	0.001	0.16
natural	4.1	3.8	3.4	1.2	1.2	1.0	0.995	2	80	8.60	0.000	0.18
open	3.5	3.1	3.2	1.1	0.9	0.8	0.933	2	80	3.19	0.050	0.07
sad	2.7	2.2	1.9	1.4	1.2	1.1	0.750	2	80	8.61	0.001	0.18

d: downward, s: sideward, u: upward.

Table S11: Main effects of gaze behavior in the negative context group in Experiment 2

Negative	*M*			*SD*			Main Effect Gaze Behavior					
**Blink frequency**	2s	4s	6s	2s	4s	6s	ε	df1	df2	*F*	*p*	η_p^2
annoyed	3.0	2.1	2.0	1.5	1.2	1.3	0.884	2	76	7.85	0.001	0.17
attentive	4.4	5.2	5.5	1.5	1.5	1.2	0.789	2	76	10.45	0.000	0.22
bewildered	2.8	1.9	2.1	1.5	1.1	1.4	0.899	2	76	9.48	0.000	0.20
calm	3.9	5.3	5.6	1.7	1.3	1.2	0.904	2	76	17.90	0.000	0.32
empathetic	3.4	4.0	3.9	1.4	1.7	1.6	0.912	2	76	2.46	0.097	0.06
interested	3.8	4.4	4.8	1.6	1.6	1.5	0.948	2	76	5.57	0.006	0.13
likeable	3.8	4.3	4.3	1.3	1.4	1.5	0.967	2	76	2.45	0.095	0.06
natural	3.3	4.6	4.4	1.5	1.6	1.8	0.861	2	76	9.07	0.001	0.19
open	3.9	4.6	4.9	1.4	1.5	1.6	0.911	2	76	7.53	0.002	0.17
sad	2.8	3.3	2.7	1.5	1.4	1.4	0.915	2	76	3.77	0.031	0.09
**Constant gaze direction**	d	s	u	d	s	u	ε	df1	df2	*F*	*p*	η_p^2
annoyed	2.8	3.7	4.1	1.5	1.8	2.0	0.944	2	76	7.45	0.001	0.16
attentive	4.0	2.2	3.4	1.7	1.3	2.1	0.859	2	76	12.59	0.000	0.25
bewildered	2.9	2.6	2.7	1.7	1.5	1.8	0.985	2	76	0.47	0.625	0.01
calm	4.8	5.2	5.0	1.7	1.5	1.7	0.991	2	76	1.00	0.372	0.03
empathetic	3.7	2.0	2.2	1.9	1.2	1.6	0.971	2	76	18.54	0.000	0.33
interested	3.5	2.0	2.7	1.5	1.3	1.9	0.970	2	76	9.90	0.000	0.21
likeable	3.6	2.5	2.5	1.5	1.2	1.4	0.984	2	76	11.42	0.000	0.23
natural	3.7	3.1	2.9	1.6	1.4	1.5	0.953	2	76	3.94	0.025	0.09
open	3.2	1.6	2.6	1.5	0.9	1.9	0.829	2	76	12.30	0.000	0.24
sad	4.0	2.4	2.1	1.8	1.5	1.5	0.810	2	76	29.27	0.000	0.44
**Shift frequency**	2s	4s	6s	2s	4s	6s	ε	df1	df2	*F*	*p*	η_p^2
annoyed	3.7	3.4	2.9	1.2	1.3	1.2	0.951	2	76	13.57	0.000	0.26
attentive	3.4	3.4	4.3	1.2	1.1	1.0	0.937	2	76	12.93	0.000	0.25
bewildered	2.9	2.6	2.2	1.2	1.1	1.0	0.985	2	76	10.04	0.000	0.21
calm	3.1	4.1	4.8	1.1	1.2	1.0	0.850	2	76	38.47	0.000	0.50
empathetic	3.0	3.2	3.7	1.1	0.9	1.2	0.982	2	76	11.33	0.000	0.23
interested	2.9	3.1	3.8	1.0	0.8	1.0	0.983	2	76	16.51	0.000	0.30
likeable	3.2	3.3	3.7	0.9	0.7	1.0	0.980	2	76	8.59	0.000	0.18
natural	3.6	4.0	4.2	1.1	0.9	1.2	0.798	2	76	8.66	0.001	0.19
open	2.6	2.7	3.6	0.7	0.8	1.0	0.916	2	76	21.24	0.000	0.36
sad	2.7	2.8	3.1	1.3	0.9	1.2	0.997	2	76	4.72	0.012	0.11
**Shift direction**	d	s	u	d	s	u	ε	df1	df2	*F*	*p*	η_p^2
annoyed	2.6	3.6	3.8	1.2	1.5	1.4	0.958	2	76	17.84	0.000	0.32
attentive	4.6	3.3	3.2	1.0	1.0	0.9	0.947	2	76	46.78	0.000	0.55
bewildered	2.6	2.8	2.3	1.1	1.3	1.1	0.963	2	76	4.63	0.014	0.11
calm	4.3	3.6	4.2	1.1	1.2	1.1	0.996	2	76	6.02	0.004	0.14
empathetic	4.2	2.9	2.7	1.4	1.1	1.0	0.907	2	76	32.51	0.000	0.46
interested	4.2	2.9	2.7	1.1	1.0	0.9	0.913	2	76	38.52	0.000	0.50
likeable	4.2	3.2	2.8	1.1	0.8	0.8	0.856	2	76	36.54	0.000	0.49
natural	4.4	3.9	3.4	1.2	1.1	1.1	0.963	2	76	18.09	0.000	0.32
open	3.5	2.7	2.7	0.9	0.9	0.8	0.969	2	76	15.37	0.000	0.29
sad	3.9	2.3	2.3	1.5	1.1	1.1	0.961	2	76	50.39	0.000	0.57

d: downward, s: sideward, u: upward.

Table S12: Interactions between shift direction and shift frequency in Experiment 2

	Interaction Shift Direction x Shift Frequency					
	ε	df1	df2	*F*	*p*	η_p^2
annoyed	0.938	4	312	0.98	0.416	0.01
attentive	0.944	4	312	3.16	0.016	0.04
bewildered	0.891	4	312	0.47	0.738	0.01
calm	0.958	4	312	0.69	0.594	0.01
empathetic	0.902	4	312	3.23	0.016	0.04
interested	0.886	4	312	2.14	0.084	0.03
likeable	0.937	4	312	1.06	0.375	0.01
natural	0.935	4	312	3.46	0.010	0.04
open	0.931	4	312	0.41	0.787	0.01
sad	0.900	4	312	2.20	0.076	0.03

Table S13: Main effects of shift frequency separated by shift direction

	M			*SD*			Main Effect Shift Frequency					
**Down**	2s	4s	6s	2s	4s	6s	ε	df1	df2	*F*	*p*	η_p^2
annoyed	3.2	2.9	2.5	1.5	1.4	1.5	0.997	2	158	8.74	0.000	0.10
attentive	3.8	4.0	4.5	1.6	1.4	1.5	0.976	2	158	8.42	0.000	0.10
bewildered	3.0	2.7	2.3	1.6	1.5	1.3	0.996	2	158	11.03	0.000	0.12
calm	3.3	4.2	5.0	1.5	1.4	1.4	0.920	2	158	38.12	0.000	0.33
empathetic	3.6	3.8	3.9	1.5	1.6	1.6	0.977	2	158	1.65	0.197	0.02
interested	3.6	3.6	4.1	1.6	1.5	1.4	0.975	2	158	6.47	0.002	0.08
likeable	3.9	4.1	4.4	1.3	1.3	1.2	0.982	2	158	5.85	0.004	0.07
natural	3.9	4.4	4.5	1.6	1.5	1.6	0.876	2	158	4.75	0.013	0.06
open	3.1	3.4	4.0	1.2	1.3	1.5	0.938	2	158	12.70	0.000	0.14
sad	3.1	3.5	3.2	1.9	1.8	1.8	0.976	2	158	2.65	0.075	0.03
**Side**	2s	4s	6s	2s	4s	6s	ε	df1	df2	*F*	*p*	η_p^2
annoyed	4.0	3.7	3.0	1.6	1.7	1.4	0.967	2	158	17.69	0.000	0.18
attentive	2.7	3.0	3.9	1.5	1.3	1.2	0.956	2	158	25.67	0.000	0.25
bewildered	3.3	2.8	2.5	1.6	1.5	1.5	0.947	2	158	10.44	0.000	0.12
calm	2.5	3.8	4.5	1.4	1.4	1.5	0.955	2	158	54.77	0.000	0.41
empathetic	2.5	2.7	3.4	1.2	1.2	1.5	0.968	2	158	18.21	0.000	0.19
interested	2.4	2.8	3.6	1.3	1.2	1.4	0.960	2	158	24.50	0.000	0.24
likeable	3.0	3.3	3.8	1.2	1.2	1.2	0.982	2	158	12.42	0.000	0.14
natural	3.4	3.9	4.2	1.4	1.3	1.5	0.978	2	158	14.04	0.000	0.15
open	2.4	2.8	3.4	1.1	1.2	1.3	0.973	2	158	20.98	0.000	0.21
sad	2.2	2.2	2.3	1.3	1.4	1.4	0.928	2	158	0.55	0.566	0.01
**Up**	2s	4s	6s	2s	4s	6s	ε	df1	df2	*F*	*p*	η_p^2
annoyed	4.3	3.8	3.3	1.6	1.6	1.6	0.948	2	158	15.46	0.000	0.16
attentive	2.5	3.2	3.9	1.4	1.4	1.3	0.971	2	158	26.72	0.000	0.25
bewildered	3.0	2.7	2.2	1.5	1.6	1.3	0.968	2	158	16.80	0.000	0.18
calm	3.0	4.1	4.9	1.5	1.5	1.2	1.000	2	158	53.13	0.000	0.40
empathetic	2.4	2.7	3.4	1.3	1.1	1.4	0.968	2	158	18.51	0.000	0.19
interested	2.3	2.7	3.4	1.3	1.2	1.3	0.957	2	158	19.13	0.000	0.19
likeable	2.8	3.0	3.6	1.2	1.1	1.3	0.970	2	158	14.82	0.000	0.16
natural	2.7	3.5	4.1	1.4	1.4	1.4	0.951	2	158	29.23	0.000	0.27
open	2.4	2.9	3.5	1.2	1.0	1.2	0.897	2	158	26.62	0.000	0.25
sad	2.1	2.0	2.2	1.3	1.2	1.4	0.955	2	158	1.50	0.227	0.02

Table S14: Overview of Principal Component Analyses

Valence	Condition	KMO	n of PC	PC1 var(%)	PC2 var(%)	PC3 var(%)	Correl. PC1-PC2	Correl. PC1-PC3	Correl. PC2-PC3
neutral	Blink_2s	0.813	2	0.55	0.13	-	0.05	-	-
neutral	Blink_4s	0.788	2	0.40	0.23	-	0.35	-	-
neutral	Blink_6s	0.789	2	0.41	0.23	-	-0.31	-	-
neutral	Constant_down	0.776	2	0.42	0.19	-	-0.10	-	-
neutral	Constant_side	0.755	3	0.38	0.18	0.13	-0.01	0.19	-0.08
neutral	Constant_up	0.889	2	0.54	0.17	-	-0.17	-	-
neutral	Shift_down_2s	0.774	2	0.50	0.16	-	-0.20	-	-
neutral	Shift_down_4s	0.806	3	0.35	0.24	0.14	-0.42	0.20	-0.07
neutral	Shift_down_6s	0.747	3	0.33	0.19	0.15	-0.18	0.34	-0.14
neutral	Shift_side_2s	0.743	3	0.43	0.15	0.13	-0.17	0.20	0.00
neutral	Shift_side_4s	0.814	2	0.50	0.14	-	-0.19	-	-
neutral	Shift_side_6s	0.786	2	0.41	0.20	-	-0.21	-	-
neutral	Shift_up_2s	0.859	2	0.54	0.16	-	-0.01	-	-
neutral	Shift_up_4s	0.814	3	0.41	0.14	0.16	0.38	0.09	0.05
neutral	Shift_up_6s	0.818	2	0.53	0.19	-	-0.21	-	-
negative	Blink_2s	0.725	2	0.41	0.27	-	-0.08	-	-
negative	Blink_4s	0.800	3	0.52	0.17	0.13	0.02	-0.10	0.11
negative	Blink_6s	0.729	2	0.43	0.23	-	-0.11	-	-
negative	Constant_down	0.747	3	0.43	0.19	0.15	0.08	0.29	0.04
negative	Constant_side	0.747	3	0.42	0.18	0.13	-0.01	0.10	-0.01
negative	Constant_up	0.839	2	0.54	0.19	-	0.00	-	-
negative	Shift_down_2s	0.760	2	0.44	0.21	-	0.14	-	-
negative	Shift_down_4s	0.779	2	0.47	0.23	-	-0.08	-	-
negative	Shift_down_6s	0.793	2	0.45	0.23	-	-0.11	-	-
negative	Shift_side_2s	0.753	3	0.44	0.16	0.14	-0.05	0.07	0.19
negative	Shift_side_4s	0.722	3	0.43	0.18	0.13	0.04	0.03	0.12
negative	Shift_side_6s	0.788	3	0.41	0.17	0.14	0.08	-0.18	-0.18
negative	Shift_up_2s	0.760	3	0.43	0.13	0.11	-0.03	0.02	0.05
negative	Shift_up_4s	0.813	3	0.46	0.15	0.13	0.09	0.08	-0.06
negative	Shift_up_6s	0.719	2	0.44	0.21	-	-0.17	-	-

KMO: Kaiser-Meyer-Olkin measure. PC: principial component. n of PC: number of components extracted according to the Kaiser criterion (eigenvalues > 1). var(%): proportion of variance. Correl: correlation.

Table S15: Factor loadings of all 30 Principal Component Analyses

		Neutral			Negative		
**Condition**	**Category**	**PC1**	**PC2**	**PC3**	**PC1**	**PC2**	**PC3**
Blink_2s	annoyed	-0.84	0.08	-	-0.24	0.71	-
	attentive	0.65	0.24	-	0.68	-0.47	-
	bewildered	-0.71	-0.12	-	-0.10	0.80	-
	calm	0.41	0.78	-	-0.16	-0.77	-
	empathetic	0.73	-0.20	-	0.87	0.30	-
	interested	0.91	-0.01	-	0.90	0.06	-
	likeable	0.84	0.14	-	0.88	0.07	-
	natural	0.82	0.11	-	0.63	-0.25	-
	open	0.85	-0.15	-	0.77	-0.37	-
	sad	-0.44	0.74	-	0.44	0.65	-
Blink_4s	annoyed	-0.40	-0.39	-	-0.19	0.79	0.18
	attentive	0.57	0.40	-	0.80	0.17	-0.30
	bewildered	-0.09	-0.81	-	-0.07	-0.01	0.95
	calm	-0.15	0.84	-	-0.05	-0.93	0.11
	empathetic	0.91	-0.08	-	0.88	0.05	-0.03
	interested	0.96	-0.12	-	0.96	0.00	-0.03
	likeable	0.93	0.01	-	0.90	-0.06	-0.08
	natural	0.52	0.31	-	0.88	-0.23	0.17
	open	0.62	0.38	-	0.86	-0.07	-0.01
	sad	-0.09	-0.35	-	0.65	0.34	0.42
Blink_6s	annoyed	-0.46	0.60	-	-0.25	0.61	-
	attentive	0.77	-0.14	-	0.67	-0.32	-
	bewildered	-0.27	0.51	-	-0.30	0.75	-
	calm	-0.05	-0.78	-	-0.06	-0.79	-
	empathetic	0.91	0.17	-	0.90	0.22	-
	interested	0.94	0.12	-	0.79	-0.03	-
	likeable	0.70	-0.03	-	0.91	-0.01	-
	natural	0.67	-0.31	-	0.81	-0.04	-
	open	0.57	-0.37	-	0.82	-0.09	-
	sad	0.16	0.79	-	0.35	0.74	-
Constant_down	annoyed	-0.30	0.43	-	-0.33	0.65	0.07
	attentive	0.79	-0.07	-	0.60	-0.18	0.52
	bewildered	0.05	0.77	-	0.03	0.85	0.06
	calm	-0.07	-0.82	-	-0.28	-0.74	-0.06
	empathetic	0.91	0.15	-	0.96	0.03	-0.04
	interested	0.91	0.02	-	0.74	-0.13	0.33
	likeable	0.86	0.15	-	0.81	0.13	0.18
	natural	0.57	-0.27	-	0.88	-0.14	-0.12

		Neutral			Negative		
	open	0.82	-0.23	-	-0.04	0.12	0.95
	sad	-0.27	0.51	-	0.84	0.29	-0.18
Constant_side	annoyed	-0.36	0.61	0.29	-0.44	0.38	0.68
	attentive	0.88	-0.19	0.01	0.81	-0.12	-0.13
	bewildered	0.12	0.72	-0.27	-0.10	0.86	-0.16
	calm	-0.25	-0.50	0.44	-0.32	-0.74	-0.12
	empathetic	0.83	0.01	0.03	0.85	0.06	0.06
	interested	0.91	0.10	0.05	0.91	0.06	-0.08
	likeable	0.69	0.05	0.44	0.77	-0.04	0.29
	natural	0.18	0.00	0.85	0.27	-0.27	0.81
	open	0.88	0.03	-0.09	0.70	-0.15	0.12
	sad	-0.03	0.79	0.11	0.73	0.49	-0.01
Constant_up	annoyed	-0.73	0.26	-	-0.67	0.35	-
	attentive	0.89	0.01	-	0.86	-0.16	-
	bewildered	0.03	0.83	-	-0.07	0.74	-
	calm	0.15	-0.80	-	0.18	-0.83	-
	empathetic	0.89	0.08	-	0.80	0.46	-
	interested	0.96	0.01	-	0.91	-0.03	-
	likeable	0.89	-0.09	-	0.86	-0.08	-
	natural	0.75	-0.05	-	0.71	-0.24	-
	open	0.95	0.09	-	0.92	-0.11	-
	sad	0.32	0.56	-	0.75	0.50	-
Shift_down_2s	annoyed	-0.73	0.38	-	-0.11	-0.75	-
	attentive	0.79	0.18	-	0.43	0.45	-
	bewildered	-0.19	0.73	-	0.22	-0.60	-
	calm	0.57	0.04	-	-0.37	0.66	-
	empathetic	0.84	0.11	-	0.91	0.01	-
	interested	0.83	0.09	-	0.90	0.10	-
	likeable	0.86	-0.01	-	0.91	0.05	-
	natural	0.79	-0.21	-	0.84	-0.10	-
	open	0.83	0.03	-	0.29	0.68	-
	sad	0.11	0.90	-	0.85	-0.07	-
Shift_down_4s	annoyed	-0.04	0.86	-0.02	-0.08	0.80	-
	attentive	0.61	-0.30	0.16	0.62	-0.42	-
	bewildered	0.15	0.82	0.25	0.10	0.81	-
	calm	0.15	-0.73	0.09	-0.07	-0.75	-
	empathetic	0.52	-0.18	0.51	0.89	0.05	-
	interested	0.71	-0.08	0.18	0.83	-0.22	-
	likeable	0.93	0.03	-0.15	0.94	0.17	-
	natural	0.88	0.14	-0.14	0.79	0.05	-
	open	0.62	-0.36	0.17	0.73	-0.30	-

		Neutral			Negative		
	sad	-0.11	0.11	0.93	0.85	0.19	-
Shift_down_6s	annoyed	-0.46	0.35	-0.09	-0.04	0.87	-
	attentive	0.84	-0.13	-0.02	0.71	-0.11	-
	bewildered	-0.06	0.77	0.03	0.23	0.83	-
	calm	-0.10	-0.70	0.34	0.25	-0.81	-
	empathetic	0.70	0.12	0.26	0.87	-0.06	-
	interested	0.92	0.02	-0.07	0.81	-0.16	-
	likeable	0.50	0.11	0.57	0.90	0.13	-
	natural	-0.02	-0.06	0.93	0.86	-0.14	-
	open	0.80	-0.04	-0.05	0.34	-0.24	-
	sad	-0.09	0.77	0.13	0.87	0.23	-
Shift_side_2s	annoyed	-0.59	0.48	0.22	-0.25	0.20	0.75
	attentive	0.89	0.09	0.06	0.81	-0.12	0.01
	bewildered	0.01	0.83	-0.35	0.31	0.85	0.03
	calm	0.31	0.05	0.30	0.35	-0.70	0.05
	empathetic	0.89	0.13	0.10	0.89	0.07	0.16
	interested	0.96	-0.04	-0.03	0.86	-0.01	0.03
	likeable	0.64	-0.19	0.15	0.89	0.13	-0.13
	natural	0.08	-0.09	0.87	0.35	-0.36	0.68
	open	0.94	0.01	-0.05	0.75	-0.20	-0.43
	sad	0.07	0.70	0.40	0.67	0.32	0.37
Shift_side_4s	annoyed	-0.59	0.23	-	-0.46	0.19	0.61
	attentive	0.85	0.01	-	0.82	0.07	-0.22
	bewildered	-0.29	0.67	-	-0.06	0.92	-0.04
	calm	0.55	0.13	-	0.32	-0.53	-0.34
	empathetic	0.84	0.01	-	0.91	0.06	0.14
	interested	0.92	-0.02	-	0.92	-0.04	-0.04
	likeable	0.83	-0.03	-	0.87	0.06	0.06
	natural	0.71	-0.21	-	0.31	-0.09	0.82
	open	0.84	0.19	-	0.77	-0.04	0.26
	sad	0.10	0.90	-	0.41	0.76	-0.05
Shift_side_6s	annoyed	-0.49	0.25	-	-0.04	0.05	0.92
	attentive	0.51	-0.12	-	0.78	0.35	-0.11
	bewildered	-0.07	0.87	-	0.30	-0.79	0.24
	calm	0.41	-0.55	-	0.34	0.81	0.16
	empathetic	0.86	0.26	-	0.87	-0.15	-0.15
	interested	0.87	0.05	-	0.87	-0.01	-0.06
	likeable	0.81	-0.07	-	0.80	0.07	-0.03
	natural	0.76	-0.07	-	0.66	0.14	0.16
	open	0.76	-0.19	-	0.38	0.18	-0.58
	sad	0.14	0.83	-	0.69	-0.43	0.10

		Neutral			Negative		
Shift_up_2s	annoyed	-0.72	0.35	-	-0.13	-0.08	0.88
	attentive	0.82	0.06	-	0.74	0.45	0.05
	bewildered	-0.20	0.72	-	0.60	-0.39	-0.09
	calm	0.65	-0.28	-	-0.07	0.92	-0.05
	empathetic	0.88	0.27	-	0.89	-0.03	0.03
	interested	0.91	0.10	-	0.89	0.00	-0.12
	likeable	0.83	-0.12	-	0.79	0.02	-0.06
	natural	0.86	0.18	-	0.53	0.13	0.52
	open	0.83	-0.24	-	0.51	-0.02	0.26
	sad	0.18	0.83	-	0.82	-0.24	-0.02
Shift_up_4s	annoyed	0.06	-0.87	0.16	-0.56	-0.01	0.64
	attentive	0.80	0.07	-0.08	0.69	0.46	-0.10
	bewildered	-0.39	-0.07	0.73	0.16	-0.51	0.61
	calm	0.15	0.67	0.19	0.01	0.91	0.02
	empathetic	0.67	0.37	0.09	0.86	0.02	0.03
	interested	0.77	0.20	0.17	0.91	-0.11	-0.06
	likeable	0.69	0.09	0.19	0.83	0.11	0.24
	natural	0.87	-0.15	-0.10	0.46	0.21	0.63
	open	0.92	-0.11	-0.04	0.83	0.11	0.07
	sad	0.21	-0.02	0.85	0.80	-0.38	-0.09
Shift_up_6s	annoyed	-0.77	0.11	-	-0.27	0.65	-
	attentive	0.84	-0.03	-	0.49	-0.54	-
	bewildered	-0.07	0.82	-	0.16	0.85	-
	calm	0.49	-0.59	-	0.06	-0.58	-
	empathetic	0.88	0.07	-	0.90	0.05	-
	interested	0.88	-0.06	-	0.90	-0.15	-
	likeable	0.85	0.16	-	0.78	-0.20	-
	natural	0.70	-0.29	-	0.75	0.16	-
	open	0.93	0.11	-	0.69	-0.26	-
	sad	0.20	0.82	-	0.85	0.33	-

PC: principial component.
